# A review of Chinese medicine for the treatment of psoriasis: principles, methods and analysis

**DOI:** 10.1186/s13020-021-00550-y

**Published:** 2021-12-20

**Authors:** Yang Su, Wen Qin, Lun Wu, Bingyou Yang, Qiuhong Wang, Haixue Kuang, Genhong Cheng

**Affiliations:** 1 School of Pharmacy, Heilongjiang University of Chinese Medicine, Harbin, China; 2 Institute of Chinese Medicine, Heilongjiang University of Chinese Medicine, Harbin, China; 3 School of Chinese Medicine, Guangdong Pharmaceutical University, Guangzhou, China; 4 Faculty of Microbiology and Immunogenetics, University of California, Los Angeles, CA USA

**Keywords:** Psoriasis, Chinese medicine, Syndrome type, Etiology and pathogenesis, Treatment

## Abstract

Chinese medicine (CM) has a long history in the treatment of psoriasis, and its different understanding angle and treatment mode from modern medicine have unique wisdom. In this article, we consulted a large number of literatures and collected data in order to systematically clarify how CM understands psoriasis, including the classification and judgment of syndrome types, the diagnosis and thinking of etiology and pathogenesis, the application of treatment ideas, principles, methods and various prescriptions, and suggestions on patient care, so as to provide a clear CM treatment process and experience for the world clinic. In addition, we analyzed the efficacy and safety through clinical data, and summarize the methods and prescriptions worthy of promotion, so as to provide reference for doctors to prescribe prescriptions and medication. Finally, we found that compared with modern medicine, CM has unique advantages in the treatment of psoriasis, including the uniqueness of syndrome classification, the abundantly and flexibility of treatment ideas. Not only many famous doctors have studied the ideas in ancient books, but also made great efforts to innovate, which has left a lot of valuable experiences for clinical practice. Moreover, a large number of treatment methods, prescriptions and medicines have achieved high effectiveness and safety in clinical practices for many years, which has been proved to be worthy of clinical reference and in-depth research. In addition, we also think about the deficiencies of CM in the treatment of psoriasis and the direction of breakthrough combined with modern medicine. The treatment of psoriasis with CM is worth popularizing, and we hope it can benefit more patients.

**Supplementary Information:**

The online version contains supplementary material available at 10.1186/s13020-021-00550-y.

## Background

Psoriasis has always been a difficult disease that plagues the world. At present, both CM and modern medicine have studied psoriasis from different angles, including syndrome classification, pathogenesis, treatment principles, ideas and methods. Modern medicine divides psoriasis into four types: vulgaris type, pustular type, erythroderma type and arthropathy type [1]. And it is considered to be an immune abnormal chronic inflammatory proliferative skin disease determined by multiple genes and stimulated by multiple environmental factors [2]. At present, the widely recognized pathogenesis includes Th1 and Th17 mediated chronic inflammatory response, epidermal hyperplasia and chronic neoangiogenesis [3]. Therefore, modern medicine adheres to the principles of regulating immunity, inhibiting excessive keratinization of epidermal cells and anti-inflammatory, and carries out treatment through immunosuppressants, retinoic acid preparations, biological agents, phototherapy and so on [4, 5]. These methods have made great contributions to clinical practice, but they also face some limitations, such as unsatisfactory effect, side effects, drug dependence and so on [6].

So, what are the achievements of CM in the exploration of psoriasis? In this regard, we learned about the treatment ideas and clinical status of many doctors from ancient times to now by consulting the literature. At the same time, we were pleasantly surprised to find that CM does have certain advantages in this field. It is worth mentioning that CM has a long history of treating psoriasis. By consulting the Chinese version of China National Knowledge Infrastructure (CNKI) (https://www.cnki.net/), we found that the earliest literature on the treatment of psoriasis with Chinese medicine was published in 1957 [7]. Then, the number of such literature increased rapidly, and the efficiency and security were very high. Moreover, a total of 2376 literatures were retrieved from the results of a statistical study on this type of literature carried out in 2019 [8]. For CM people, these are very valuable wealth. Therefore, starting with the classification, pathogenesis and clinical characteristics of psoriasis in CM, this paper further summarizes the treatment principles and ideas, and summarizes and analyzes the methods with high efficiency and safety, in order to let the world has some understanding of the experience of CM in the treatment of psoriasis, and provide more ideas and methods for future clinical practice. Of course, the treatment of psoriasis by CM also faces some obstacles and challenges. In this regard, we have also discussed, in order to provide some more in-depth analysis on the feasibility of the treatment discussed in this paper, and to make a breakthrough in this field of CM.

## Classification of psoriasis syndrome types in CM

At present, CM classifies psoriasis into four types (Four-Type): Blood Heat type, Blood Stasis type, Blood Dryness type and Blood Deficiency type.

First, Four-Type is the result of progress in the long historical development. Initially, in the Sui and Tang Dynasties, doctors believed that psoriasis was caused by pathogenic factors in the external environment, such as wind, heat, cold, dryness and humidity, and did not take into account internal factors of the body, such as blood, viscera. Therefore, they only targeted the skin for external treatment [9]. Then, in the Jin and Yuan Dynasties, doctors gradually took into account the internal factors of the body. Not only the heat inside the body is considered by doctors, but also the theory that viscera feeling pathogenic factors can cause psoriasis was widely accepted [9]. Later, in the Ming and Qing Dynasties, doctors had a deeper understanding of psoriasis. They finally realized that external pathogenic factors could fuse with the blood, and the three types of Blood Heat, Blood Dryness and Blood Deficiency began to emerge [9]. Finally, in modern times, the classification of syndrome types is more perfect, and the Blood Deficiency is also summarized in it [9]. Thus, Four-Type was established. Second, Four-Type is the overall trend of patients. A survey of psoriasis syndromes in China in the past 60 years showed that there were 38 psoriasis syndromes, and Four-Type accounted for 68.55%, and the proportion showed a significant growth trend [10]. Lili Zhang [11] et al. searched literatures on the treatment of psoriasis with CM published in Chinese journals in recent ten years, and the results showed that three types (Blood Heat, Blood Stasis and Blood Dryness) appeared 242 times. Bingxu Deng [11] et al. investigated the distribution of syndrome types of 600 patients in three hospitals in Beijing, and the results showed that Blood Heat, Blood Stasis and Blood Dryness can be used as the basic syndrome types in the category of CM. Incidentally, although Blood Deficiency appears less frequently than other three types, it is still a very important type. Doctor Bohua Gu [9] highly recognized Blood Deficiency and suggested that doctors consider the characteristic of “deficiency” in treatment. Third, Four-Type has been highly recognized by the current medical community. Lihong Wang clearly mentioned in the article on the treatment of psoriasis from blood that the classification standard of Four Type has been widely agreed by dermatologists in the field of CM [11]. In addition, many scholars also take Four-Type as a reference in clinical research. For example, when researchers conducted clinical trials with *Zaocys Bubali Cornu* Decoction, they divided patients into these four types, so as to implement different medicine regimens [12].

In short, CM has sufficient theory and data to support the classification standard of psoriasis syndrome type, which is very perfect and rigorous at present. It is worthy of recognition for doctors to classify patients according to this standard at diagnosis. Moreover, the correct classification is the premise to ensure the effective treatment, and is the first step in the treatment process.

## CM pathogenesis and clinical characteristics of four-type

### Pathogenesis

**Fig. 1:**
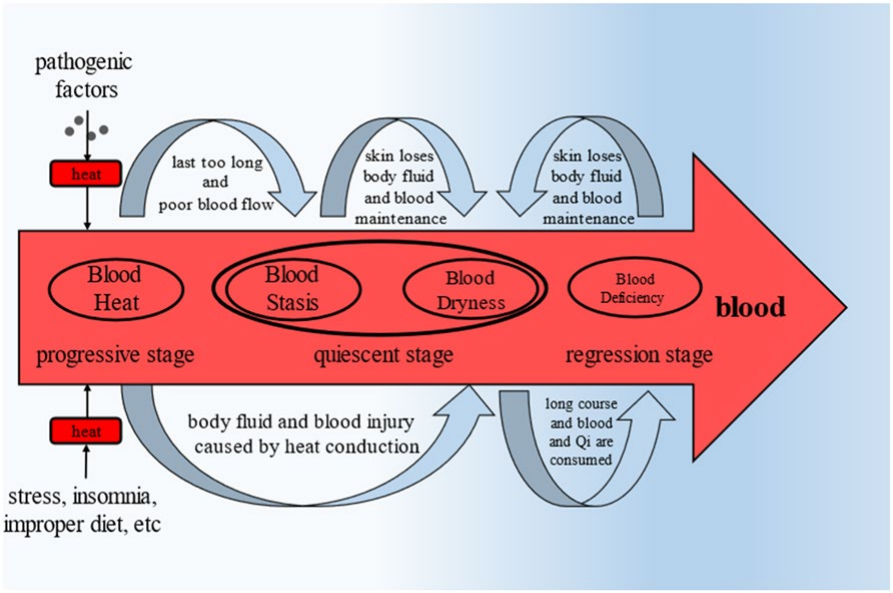
The process of onset and mutual transformation of four-type

CM also believes that four types are in order. It can be summarized as follows [13]: Blood Heat is the initial type, which is in progressive stage; Blood Stasis is the type developed from Blood Heat, which is in static period; Blood Dryness can be developed from other three types, which is in static stage and regression stage; Blood Deficiency is the type in the last stage, which is in regression stage. This conclusion can well reflect the pathogenesis, Fig. 1.

“Psoriasis is formed by the fusion of pathogenic factors in the environment and blood after invading the skin” recorded in the CM classic clearly shows that the root cause of psoriasis lies in the blood [11]. The above contents also mentioned that doctors in the Jin and Yuan Dynasties recognized that a series of reactions after the fusion of pathogenic factors and blood were important links in the process [14]. The important reaction of blood is to generate heat. Many doctors who are good at treating psoriasis from blood, such as Yanping Bai, highly agree with this theory [2]. The patient’s hot blood is blazing and stagnant in the skin, so it is regarded as Blood Heat at the beginning [2]. In addition, Bingnan Zhao believes that improper diet, terrible emotion, insomnia and other reasons will also make the body generate heat and enter the blood [15]. And because of the characteristics of heat, the incidence speed of patients in this period is very fast [15]. Then, if the patient is not treated in time, that is, the state of blood Heat lasts too long, the blood will be stagnant and the blood circulation will not be unblocked, so as to develop into Blood Stasis [15]. “Blood will form blood clots with Blood Stasis if it continues to be heat” recorded in the CM classic also expresses this meaning [16]. At the same time, due to Blood Heat and Blood Stasis, the skin will become very dry due to the loss of moisture nourishment, resulting in Blood Dryness [17]. Finally, because the course of disease is too long, the patient will consume the blood and body fluid, resulting in Blood Deficiency [15]. In addition, due to Blood Deficiency, the skin loses maintenance, resulting in Blood Dryness [17]. So, patients in this period are weak and difficult to recover [15].

In conclusion, the pathogenesis can reflect that the syndrome types of patients may change continuously, but they are not complex. Just know that if doctors ignore any link, especially the final Blood Deficiency, the patient will not recover completely. This also explains to some extent why many patients have a long course of disease and are easy to relapse. Therefore, we suggest that doctors observe and diagnose the stage at any time. For example, in the treatment of Blood Stasis, they should observe whether the patient has the symptoms of Blood Dryness and/or Blood Deficiency, so as to adjust the treatment plan. This is the key to ensure patient recovery and minimize recurrence.

### Clinical characteristics

How can doctors distinguish Four-Type? We summarized the clinical symptoms and pathogenesis characteristics on it [18–20], as shown in Table 1.

**Table 1:** The differences of the four-type

Type	Skin lesions	Tongue	Accompanied symptoms	Onset characteristics
Blood Heat	Rashes developed rapidly Shape of drop, coin, or mixture Lesions color is red Scales are ceaseless	Tongue color is red	Upset Thirsty Constipation Short and red urine	Progression stage Short course Patients have a history of mental and dietary factors, cold, tonsillitis and pharyngitis
Blood Stasis	Lesions are stiff, thick Most lesions are coins or plaques of different sizes, a few are oyster shaped Lesions color is dark red Scales are thick and dry Scales are not easy to fall off New rashes are rare Itching or not	Tongue color is dark purple tongue or dark red with ecchymosis Tongue coating is thin, white or yellow	Dry mouth but not willing to drink	Quiescent stage Long course of illness
Blood Dryness	Lesions’ shape of plaque Lots of scales Lesions’ base is bright or light red Even dry and bleeding Heat dissolves Dryness: Lesions burn the hand and pain Deficiency turns into Dryness: Base color is light and scale is not thick and sometimes with itching	Heat dissolves Dryness: Tongue color is red Tongue coating color is yellow Deficiency turns into Dryness: Tongue is pale Tongue coating is clean	Heat dissolves Dryness: Dry mouth and throat Constipation Short and red urine	Progressive or quiescent stage
Blood Deficiency	Lesions are thin Most Lesions are patchy, or all over the body Pale red or dull Scales are dry and fall off layer by layer New rashes are rare Pruritus is mild or severe	Tongue color is pale red tongue Tongue coating is less or clean	Lusterless complexion Fatigue or dizziness Sleep less Loss of appetite	Quiescence period Short course of illness

In addition to observing clinical symptoms and pathogenesis characteristics, it is also essential to ask the patient about some other situations to judge the condition. First, doctors should also fully understand geographical positions, habits and customs of patients, because these factors are often the prerequisite for the onset of patients. Such as China’s eastern coastal areas are heavily wet, patients getting sick because of damp into the body [21, 22], so as to show such symptoms of Blood Heat with damp [23]: rash mostly occurs in the armpit, groin and other parts, severe itching, accompanied by fatigue, chest tightness, poor appetite, yellow urine color, heavy feeling of lower limbs, yellow and thick tongue coating, and aggravate the disease in rainy seasons. Therefore, doctors can use the prescription of eliminating dampness and heat or add some medicines to remove dampness on the basis of the original syndrome type. For example, *Sophora flavescens*, *Atractylodes tractylodes* and *Poria cocos* Decoction with clearing damp effect, the effective rate was 93.5% [24]. Northwest areas such as Xinjiang and Qinghai in China are sandstorm all the year round and climate is dry, especially in high altitude areas, the temperature difference between day and night is large, and people like to eat spicy and greasy food, so, patients feel more pathogenic factors of wind and dryness and Blood Dryness is more serious [25, 26]. The north is dry and cold, so cold is a part of the cause, such as the “Cold Envelops Heat” mentioned in 4.2.2.3 [27]. The south is rainy and hot, or people likes to eat spicy, greasy and meat food, so patients hurt the spleen and stomach, and the dampness and heat accumulate obstruction in the body, such as ChengDu city in China [28]. Equally important, the onset or aggravation season of each patient is also different, which is often the embodiment of etiology and pathogenesis, and plays a key role in judging the condition [29]. The significance of the season of onset for treatment is mentioned in 4.2.1 and 4.2.2 below.

To sum up, the clinical symptoms and characteristics of Four-Type are obviously different, which is the first doctors observe and understand when judging the syndrome type. But some other factors can’t be ignored and thinking about these aspects can also make a great contribution to classification and treatment.

## Treatment principles and ideas of CM

### Treatment principles

After determining the syndrome type and considering the etiology and pathogenesis, the treatment can be carried out according to the following principles: “Treating from blood” is the most basic means. Blood Heat should clear away heat and cool blood, Blood Stasis should promote blood circulation and remove blood stasis, Blood Dryness should moisten the skin and relieve itching, and Blood Deficiency should nourish the body and blood [13].

### Treatment ideas

Improving blood is not only the basis of treatment, but also the ultimate goal of treatment. However, the body’s viscera, Qi (the extremely fine substances with strong vitality and continuous operation in the body, one of the basic substances that constitute the body and maintain human life activities [30]), blood and meridians have a myriad of links. If the blood is affected by other factors, such as an organ, it is difficult to cure psoriasis only by improving the blood according to the above principles. Because the blood will always be affected if the most fundamental cause is not eliminated. Therefore, it is often necessary for doctors to consider more possibilities and completely remove the factors affecting blood.

#### Treat from the viscera

**Fig. 2:**
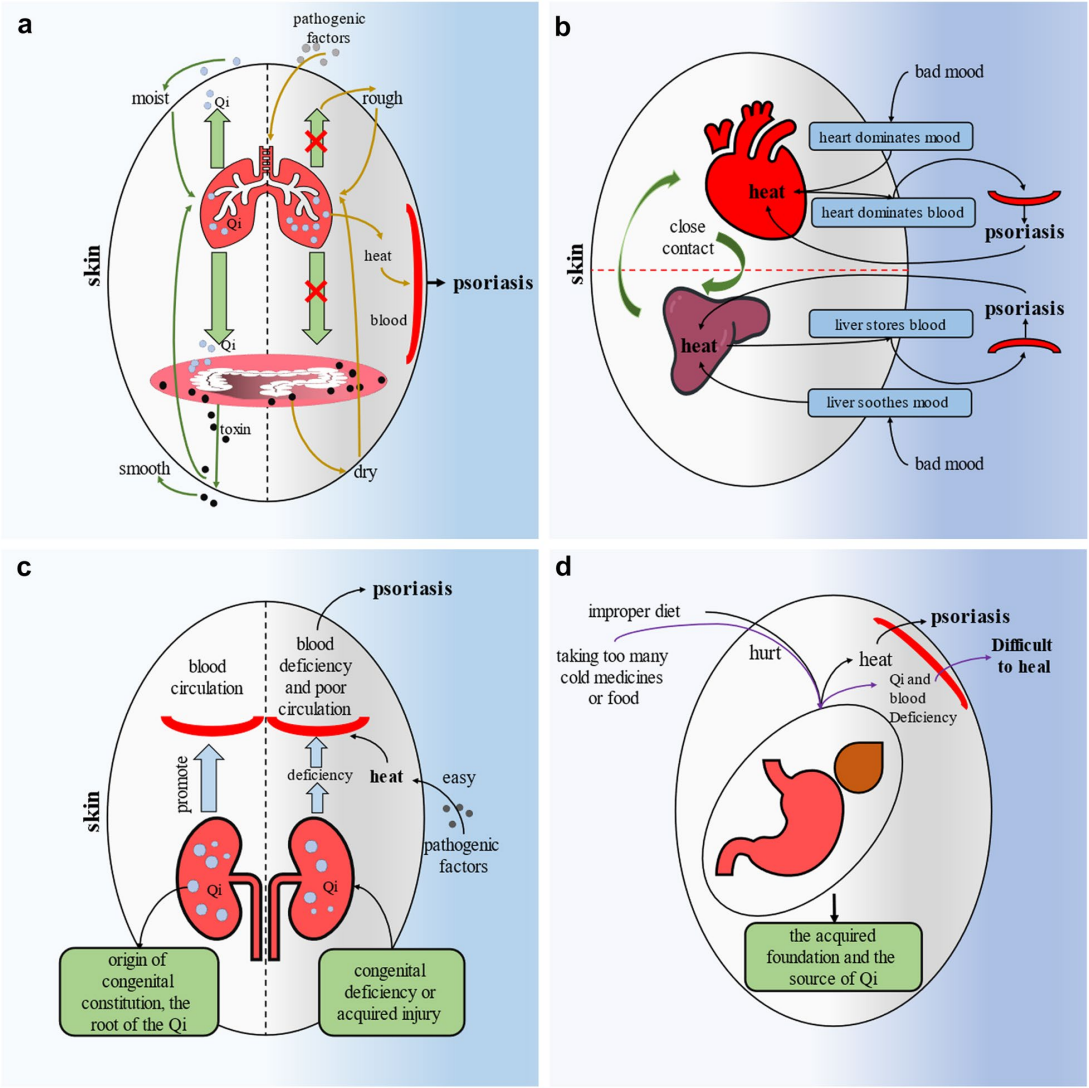
Pathogenesis from viscera

According to the experience of a large number of famous doctors discussed in the literature, we believe that viscera are the first factor that doctors should consider and often the factor that contributes the most to treatment. CM classic records [31]: “the symptoms inside must be showed outside.” Many famous modern doctors also agree that the imbalance of viscera has a role in promoting the occurrence and development of psoriasis [32]. The relationship between various organs and psoriasis is explained in detail below, and a group of diagrams are also made to illustrate it, Fig. 2. In addition, in Table 2, we also summarized the prescriptions used in the treatment of each organ according to typical cases, and the specific patient situation, treatment process and pharmacodynamic mechanism are summarized in the Additional file 1, which is very helpful for doctors’ diagnosis and medication, and the prescriptions used have no adverse reactions, and doctors can use them directly or at their discretion for suitable patients.

**Table 2:** Treatment process and prescriptions of typical cases treated from Viscera

Viscera	Treatment process	References
Lung	**Initial diagnosis:** The whole body has plaque like, drop like and map like tinea in varying degrees; There are silver white scales with uneven thickness on the surface, and scales fall off, especially on the back; Bleeding is common at the scratch of skin lesions.; The finger skin is dry and wrinkled, with thick scales and unbearable pain; Itching all over the body and more severe at night; The tongue coating is light red **Initial prescription:** *Platycodonis Radix*^1^ 90 g, *Dictamni Cortex*^5^ 60 g, 30 g of *Angelicae Sinensis Radix*^3^, *Paeoniae Radix Alba*^3^, *Rehmanniae Radix Praeparata*^3^, and *Rehmanniae Radix*^4^ respectively, *Paeoniae Radix Rubra*^4^ 20 g, *Chuanxiong Rhizoma*^3^ 20 g, 10 g of *Bombyx Batryticatus*, *Scorpio*^5^, *Zaocys*^5^, *Mori Cortex*^2^ respectively. Decoct in water and take 10 doses **Second diagnosis:** Pruritus is significantly reduced, scales are also gradually falling off, skin lesions become thinner, the color of the base of some skin lesions changes from red to light, punctate skin lesions basically subside, finger cracks are reduced, dry dandruff falls off more, and local skin is bright in color **Second prescription:** Continue to take 14 doses of the above prescription **Third diagnosis:** The skin lesions of the whole body return to normal, the finger cracks heal, and the skin is moist	[36]
Large Intestine	**Initial diagnosis:** The whole body is covered with red spots ranging from soybeans to copper coins, with severe itching; The edge of the patch is clear, and the surface is covered with silver white scales; After scraping off the scales, the film and punctate bleeding can be seen; The rash is mainly on the head, chest, back and limbs; The tongue is dark, the tip and edge of the tongue are red, and the tongue coating is thin and yellow **Initial prescription:** *Smilacis Glabrae Rhizoma* 20 g, *Angelicae Sinensis Radix*, 15 g of *Moutan Cortex*, *Salviae Miltiorrhizae Radix et Rhizoma*, *Rehmanniae Radix*, *Atractylodis Macrocephalae Rhizoma* respectively, 6 g of *Chuanxiong Rhizoma*, *Schizonepetae Herba*, *Glycyrrhizae Radix et Rhizoma* respectively, *Paeoniae Radix Alba* 30 g, *Armeniacae Semen Amarum* 10 g. Decoct in water and take 3 doses *Bombyx Batryticatus* 10 g, 2 *Bungarus Parvus*, *Scorpio* 10 g. Grind the drug into powder and divide it into 30 bags. Take 1 pack each time, twice a day **Second diagnosis:** The itching was relieved, but the skin lesions did not subside significantly **Second prescription:** Continue to take 3 doses of the above prescription **Third diagnoses:** the skin lesions still did not subside significantly **Third prescription:** Add 10 g of *Rhei Radix et Rhizoma* and 10 g of Cooked *Rhei Radix et Rhizoma* to the above prescription and take 3 doses **Forth diagnoses:** most of the skin lesions subsided, but the patient had slight abdominal pain, bowel ringing and the stool is thin and twice a day **Forth prescription:** Add 5 jujubes to the third prescription, and take 3 doses **Fifty diagnoses:** the skin lesions have basically subsided, the abdomen is no longer painful, and the stool is thin and once a day **Fifty prescription:** Continue to take the forth prescription **Finally:** the patient was basically cured (**Note:** after the patient recovered, the doctor instructed the patient to dry three times the amount of the four diagnostic prescriptions in the oven, grind them into powder and take them, 9 g each time, twice a day, two consecutive doses. Then to the next spring and winter, there was no recurrence.)	[39]
Liver	**Initial diagnosis:** Dense, coin shaped erythema and papules can be seen on the head, face, trunk and limbs, and covered with white scales. After scraping off the scales, the membrane and bleeding spots can be seen. The tongue color is red, and the tongue coating is thin and yellow **Initial prescription:** 30 g of *Bubali Cornu*^1^ (Decoct in advance), *Rehmanniae Radix*^1^, *Isatidis Radix*^3^, *Imperatae Rhizoma*^3^, *Smilacis Glabrae Rhizoma*^5^ respectively, 9 g of *Paeoniae Radix Rubra*, *Moutan Cortex*^1^, *Bupleuri Radix*^2^, *Scutellariae Radix*^2^ respectively, 15 g of *Arnebiae Radix*^3^, *Rubiae Radix et Rhizoma*^4^, *Dictamni Cortex*^5^, *Scutellariae Barbatae Herba*^3^, massa medicata fermentata^6^ () respectively, *Sophorae Flavescentis Radix* 10 g, *Glycyrrhizae Radix et Rhizoma* 6 g. Decoct in water and take 14 doses Topical Chinese medicine ointment **Second diagnosis:** There are few new rashes and less itching, but the scales are still thick. Defecate unobstructed, insomnia has also been improved **Second prescription:** The above prescriptions remove 5 and add *Salviae Miltiorrhizae Radix et Rhizoma*^7^30g, *Sappan Lignum*^7^ 9 g and *Polygoni Cuspidati Rhizoma et Radix*^7^ 30 g. Take 14 doses **Third diagnosis:** The rash began to fade, the color turned pale red, the scales became thinner, and the symptoms of poor appetite were improved **Third prescription:** Add *Spatholobi Caulis*^8^ 30 g to the prescription of the second diagnosis. Take 28 doses **Forth diagnoses:** The rash basically subsided, and only light brown pigmentation spots could be seen on the trunk and limbs, with almost no symptoms of pruritus **Forth prescription:** Remove *Bubali Cornu*, *Imperatae Rhizoma*, *Scutellariae Barbatae Herba* and add *Rehmanniae Radix Praeparata*^9^, Cooked *Polygonati Rhizoma*^9^ in the three diagnostic prescriptions. Take it for 2 weeks **Finally:** the patient was basically cured	[40]
Heart	**Initial diagnosis:** severe papules and erythema on limbs, trunk and scalp covered with scales. After scraping off the scales, the film and bleeding spots can be seen, and the itching is severe and aggravated at night. The complexion is red and the body is thin. The tongue color is dark, and the tongue coating is thin and yellow **Initial prescription:** 30 g of *Rehmanniae Radix* ^1^, *Salviae Miltiorrhizae Radix et Rhizoma*^1^, *Forsythiae Fructus*,*Hedyotis Diffusa*, *Lonice Raejaponicae Caulis*, *Polygoni Multiflori Caulis* respectively, 20 g of *Scrophulariae Radix*^1^, *Ophiopogonis Radix*, *Zaocys*^3^, *Sophorae Flavescentis Radix*,*SpatholobiCaulis* respectively,15 g of *Bubali Cornu* silk (Decoct in advance)^1^, *Lonicerae Japonicae Flos*,*Bolbostemmatis Rhizoma*, *Rumex madaio MakinoR. daiwoo Makino.*, *Bombyx Batryticatus* ^3^, *Piperis Kadsurae Caulis*, *Gleditsiae Spina*, *Astragali Radix*,*Curcumae Rhizoma*,*Sparganii Rhizoma* respectively, 10 g of *Coptidis Rhizoma*,*Lophatheri Herba* ^2^,*Cicadae Periostracum* ^3^, *Pinelliae Rhizoma Praeparatum* respectively. Decoct in water and take 21 doses **Second diagnosis:** the symptoms have improved significantly, but the rash is still red, and the itching symptoms have not been improved. And the mouth is bitter, the tongue color is dark, and the tongue coating is slightly yellow **Second prescription:** Remove *Coptidis Rhizoma*,*Lophatheri Herba*,*Cicadae Periostracum*,*Sophorae Flavescentis Radix*,*Spatholobi Caulis*, *Polygoni Multiflori Caulis*, *Gleditsiae Spina*, *Pinelliae Rhizoma Praeparatum*, *Astragali Radix*,*Curcumae Rhizoma*, *Sparganii Rhizoma*,and change *Scrophulariae Radix* 30 g, *Bubali Cornu* silk (Decoct in advance) 30 g,*Ophiopogonis Radix* 15 g, and add *Vespae Nidus* ^3^6 g, *Gastrodiae Rhizoma* ^4^10 g, *Dictamni Cortex* ^3^15 g,*Tribuli Fructus* ^4^20 g,*Saigae Tataricae Cornu* Powder^5^ (take after mixing it with water) 0.6 g. Decoct in water and take 21 doses **Third diagnosis:** The skin lesions of limbs and head were significantly improved, and the pruritus was significantly reduced, but there were symptoms of diarrhea and yellow urine. The tongue color is dim, and the tongue coating is yellow and greasy **Third prescription:** Change the dose of some medicines in the second prescription: *Bubali Cornu* silk (Decoct in advance) 30 g, *Bolbostemmatis Rhizoma* 20 g, *Vespae Nidus* 10 g Add *Scutellariae Radix* ^1^15 g, *Talcum* (Packed separately)^1^20 g Decoct in water and take 21 doses **Forth diagnoses:** The symptoms of skin lesions and pruritus basically disappeared, only a few scattered punctate rashes, occasional diarrhea and yellow urine. The tongue color is dim, and the tongue coating is yellow, greasy and slightly rough. The patient's mental state improved greatly and his confidence increased **Forth prescription:** Remove *Lonicerae Japonicae Flos*^1^ and add Fried *Atractylodis Macrocephalae Rhizoma*^6^15 g, *Paridis Rhizoma*^6^12 g in third prescriptions. Decoct in water and take 21 doses	[49]
Kidney	**Initial diagnosis:** thick skin lesions on the head, more desquamation and severe pruritus. Scattered patchy skin lesions appear on the trunk and small limbs, with white dandruff, most scratches and blood scabs on the surface. The base of the lesion is thick and dark red. The larger one is the size of a palm and the smaller one is the size of a coin. The skin lesions and tongue are dark red with ecchymosis, and the tongue coating is thin and white **Initial prescription:** *Angelicae Sinensis Radix*^2^ 12 g, *Paeoniae Radix Rubra*^2^ 12 g, *Chuanxiong Rhizoma*^2^ 10 g, 15 g of *Persicae Semen*^2^, *Carthami Flos*^2^, *Ginseng Radix Et Rhizoma* ^1^, *Citri Reticulatae Pericarpium* respectively, 30 g of *Aconiti Lateralis Radix Praeparata* ^1^9 g, *Spatholobi Caulis*^2^, *Cinnamomi Cortex* ^1^, *Glycyrrhizae Radix et Rhizoma* respectively. Decoct in water and take 4 weeks **Second diagnosis:** The skin lesions basically subsided, with only a few papules on the back, only brown pigment spots left at the regression, and sometimes dry mouth **Second prescription:** Remove *Ginseng Radix et Rhizoma*, *Aconiti Lateralis Radix Praeparata* and add 15 g of *Zingiberis Rhizoma*^3^, *Pseudostellariae Radix*^3^, *Ophiopogonis Radix*^4^ in above predcription. Decoct in water and take more than 20 doses **Third diagnosis:** The skin lesions obviously subsided to normal, with only a small of pigmented spots left **Third prescription:** Take the pills prepared by the two prescriptions until you recover	[43]
Spleen and stomach	**Initial diagnosis:** The patient has a strong body, a drop of desquamation on his chest, back and abdomen, slight itching, red and swollen lips, red tongue, yellow and thick tongue coating, yellow urine, dry stool and once every 3 ~ 4 day **Initial prescription:** *Pogostemonis Herba* ^4^30 g, *Lilii Bulbus* ^5^30 g, Fried *Gardeniae Fructus* ^1^15 g,*Rhei Radix et Rhizoma* ^1^15 g, *Saposhnikoviae Radix* ^2^75 g,*Gypsum Fibrosum* ^1^45 g, *Glycyrrhizae Radix et Rhizoma* ^6^45 g,*Moutan Cortex* ^3^45 g。Except for 3, the other drugs were crushed into coarse powder and fried with yellow rice wine; 3 grind and mix with the coarse powder of other drugs. Take 10 g daily, fry into half a bowl with water, and take it twice in the morning and evening Cassitol ointment was reduced from twice a day to once a day. It was stopped after one week **Second diagnosis:** After a week, the stool is unobstructed, twice a day. The symptoms of lip swelling disappeared, and desquamation and pruritus were not aggravated **Second prescription:** Discontinue Cassitol ointment and continue to take the above Decoction for one month **Third diagnosis:** The skin desquamation completely disappeared without pruritus, and only scattered hypopigmentation spots were seen **Finally:** the patient was followed up for half a year and the condition was stable	[50]

##### Treat from lung

CM believes that the lung dominates the skin [33]. The lung transports nutrients such as body fluid through the function of transmitting Qi upward and downward, and distributes nutrients to the skin, so as to maintain and moisturize the skin [34–36]. Lung Qi can also control the opening of sweat holes and help the skin resist external pathogenic factors [34]. In addition, the lung and skin are coordinated in physiological function. First of all, the pores on the skin can help the lung to transmit Qi through the function of dispersing Qi. Therefore, the skin can cooperate with the lung to jointly control the body’s respiratory function [34]. Secondly, the function of lung transporting Qi is very important for body fluid metabolism, and the skin controls perspiration by opening and closing sweat pores, which also plays a coordinating role in the process of water metabolism [34].

Lung is a delicate organ, which is easy to be invaded by external pathogenic factors, and pathogenic factors enter from the mouth and nose and invades the lung first [34]. First, after the lung is injured by external pathogenic factors, the function of transmitting Qi upward and downward is affected, so that nutrients cannot be transported to the skin [34, 37]. Therefore, the skin will become dry due to the loss of moisture and nourishment [34]. Second, the lung’s function of transporting body fluid is damaged, and the body fluid in the lung cannot circulate, resulting in heat and entering the blood due to the deposition of body fluid [34]. In addition, the lung become dry due to heat and is unable to provide moisture for the skin [34]. It is worth mentioning that in CM, autumn is the season corresponding to the lung and has the characteristics of dry climate. Therefore, psoriasis caused by the lung usually occurs or worsens in autumn [34]. Third, the long-term imbalance of the function of transporting body fluid will consume the water and other nutrients of the body, so the body will become very weak, and eventually lead to psoriasis, which is difficult to recover [34, 35].

Therefore, the doctor should consider treatment from the lung according basic principle of unblock lung Qi if patient has the following symptoms: Occurs or worsens in autumn; Often have colds before onset; The whole body has a red rash with dry throat, sore throat, red tongue and yellow tongue coating; Deterioration of respiratory function; Dysregulation of body fluid circulation [32].

##### Treat from large intestine

CM believes that the lung is closely related to the large intestine [33]. It is recorded in CM classic that “the reason why the large intestine can conduct is that lung Qi can transmit downward” [38]. The function of lung Qi downward transmission can transmit body fluid to the large intestine, so as to help the large intestine conduct and make the stool unobstructed, and discharge the toxin out of the body in time, which is conducive to moisturizing and smoothing the skin [38]. On the contrary, if the function of lung Qi downward transmission is affected, it will not be able to transport body fluid to the large intestine, so that the conduction function of the large intestine will be affected, which will not only fail to expel toxins, but also produce heat [38]. Moreover, lesions in the large intestine will also affect the function of the lung and involve the skin [38].

Therefore, the doctor should consider treatment from the large intestine according basic principle of improve the excretory function of large intestine if patient has the following symptoms: Rashes are red or dark red; Itching is severe; Dry stool; Red tongue and yellow tongue coating [38, 39].

##### Treat from liver

CM believes that the liver is responsible for soothing emotions and storing blood [33]. Therefore, the liver, blood and skin are closely related. First, the liver’s function of storing blood will be affected, which will lead to the deficiency of blood, so that the skin will lose blood nourishment, not only become dry, but also produce the symptoms of Blood Deficiency [40]. In other words, the etiology of psoriasis lies in the blood, and the liver is the organ that stores blood. Therefore, the interaction between blood and liver is endless. Second, the liver is an organ to dredge emotions. People’s emotional disorder due to excessive pressure, irritable and other will affect the function of the liver, so that the liver Qi accumulates and stagnates and produces heat [40]. In addition, the liver will also be dredged too much because the mood is too bad, so that too much liver Qi will be evacuated from all parts, leading to blood overflow outside the blood vessels, resulting in the symptoms of “bleeding points” [41].

Therefore, the doctor should consider treatment from the liver according basic principle of dredge liver Qi if patient has the following symptoms: Emotional disorders; If it occurs repeatedly, the rash shows dark red plaque; Dry scales; Bleeding points [32].

##### Treating from heart

If liver is the organ that stores blood, then heart is the organ that commands the blood and is responsible for the operation of the blood [33]. In addition, heart dominates emotion, which is closely related to the function of liver to dredge emotion [33]. If the heart function is normal, Qi can be sufficient to maintain the whole body [42]. Most importantly, CM classics also record that the Qi in the heart can be transported to the skin, and the symptoms such as pain, pruritus and sores on the skin belong to heart diseases [32]. It can be seen that the relationship between heart and skin can’t be ignored. Emotional disorders and other reasons lead to heat in the heart, and the heart is the organ that commands the blood, so the blood will also produce heat [32, 42]. And the heart damage will lead to the disordered operation of the vein, which will lead to the disordered operation of hot blood and corrode the skin [32].

Therefore, the doctor should consider treatment from the heart according basic principle of clear heat in the heart if patient has the following symptoms: Red rashes; obvious itching; Dry mouth and like drinking water; Upset; Hot urination and dry stool; Red tongue and yellow tongue coating [32].

##### Treat from kidney

CM believes that the kidney being the origin of congenital constitution, the root of the Qi [33]. The strong kidney function can not only make the body fluid and blood sufficient, but also make the blood flow more smoothly [32]. If the patient is ill for a long time, the Qi is damaged, resulting in kidney Qi deficiency, or the congenital endowment is insufficient, and the kidney Qi is weak, it is easy for the pathogenic factors to enter the body, or the kidney Qi deficiency due to sexual excess, which is easy to weaken the body and lead to the occurrence of psoriasis [43, 44]. Moreover, Qi is the commander of blood [33]. If the vital energy is insufficient, the blood will lose its command and stay to stagnate [45]. Therefore, no matter what method is used to treat psoriasis, it should always be accompanied by warming kidney and nourishing Qi to strengthen the body. Especially in the late stage of treatment, don’t always clear heat and cool blood to hurt healthy Qi [46]. Qi is not only the best medicine for curing diseases, but also the strongest defense against diseases. It is so-called “Qi exists inside, pathogenic factors can’t invade” [46].

Therefore, the doctor should consider treatment from the kidney according basic principle of strengthen kidney Qi if patient has the following conditions: Long course of disease; Pain and weak waist and back; Afraid of cold; If it occurs repeatedly, the rash is dark red spots; Dry scales; Dry mouth and throat; Red tongue, less and pale tongue coating [32].

##### Treat from spleen and stomach

If the kidney is the innate foundation and the root of Qi, then the spleen and stomach is the acquired foundation and the source of Qi [33]. If the spleen and stomach are damaged, the metaplasia of Qi and blood will lose its source. The body will not only become weak and vulnerable to pathogenic factors, but also the skin will become worse and worse due to the loss of nourishment of body fluid and blood [32]. The above also mentioned that improper diet will increase the burden on the spleen and stomach, which will turn into heat [15]. Moreover, CM believes that the spleen is an organ that transforms nutrients [33]. If this function is dysfunctional, it will produce damp due to the accumulation of body fluid and other substances, and then produce the symptoms of Damp Heat, especially the elderly and children [47, 48]. Equally important, eating food or taking medicines that are too cold can also cause damage to the spleen and stomach [48].

Therefore, the doctor should consider treatment from the spleen and stomach according basic principle of maintenance of spleen and stomach if patient has the following conditions: Poor digestive function; The diet is too sweet, greasy and cold; Obesity. It is worth emphasizing that doctors should not only judge whether psoriasis is caused by the spleen and stomach at the time of diagnosis, but also take care of the patient’s spleen and stomach at any stage of treatment [48]. In addition to always telling patients about their diet, doctors should not ignore the key points of timely applying warm medicines in the later stage of treatment to prevent the early heat clearing and blood cooling medicines from damaging the spleen and stomach.

#### Treat from other angles

If it is still difficult to find the cause, doctors might as well try some innovative methods. For some difficult and miscellaneous psoriasis, these methods have created unexpected surprises and are worthy of clinical reference.

##### Treat from “Xuan Fu”

All the viscera have Xuan Fu, which is the way for Qi, blood and body fluid to diverge and circulate. Xuan Fu closed, then blood block, heat and toxin accumulate, resulting in the onset of disease [51]. In summer, the skin pores open fully and the Qi and blood are unobstructed, so this type of patients is serious in winter and relieve in summer. In addition, due to the wind into the body is not be divergent, so before the onset, patients will appear cold, fever, nasal congestion, runny nose and other symptoms [51, 52]. In clinical practice, *Borneolum*, *Curcumae Rhizoma* are commonly used to open Xuan Fu, smooth Qi and break stasis, and at the same time, *Schisandrae Chinensis Fructus* and other astringent medicines are used to assist in the treatment of a reasonable opening and closing and not to hurt the vital Qi [52].

##### Treat from “Hidden Pathogenic Factor”

The external symptoms are cured, but the root of the disease is not removed, or the innate endowment is insufficient, resulting in the dysfunction of the viscera, and the pathogenic factors are hidden in the body, and then the disease occurs when there are certain conditions or encountering inducements, which is called “Hidden Pathogenic Factor” [53]. The body and the natural season adapt to each other, all things on earth are germinate in spring, grow in summer, harvest in autumn and store in winter, pathogenic factors are the same [53]. So, this kind of patients is serious in summer and relieve in winter, or onset in spring and summer, and relapse easily [53]. *Artemisiae Annuae Herba* and *Trionycis Carapax* Decoction is often used clinically: Medicines such as *Artemisiae Annuae Herba*, *Trionycis Carapax*, etc. to search for pathogenic factors and clear the collaterals, so that injurious Qi can reach from the inside to the outside, and it can be solved from the surface. At the same time, the medicines such as *Anemarrhenae Rhizoma* and *Rehmanniae Radix* are used to cool blood and nourish body [54].

##### Treat from “Cold Envelops Heat”

Wind-Cold is the root cause of the onset of Blood Heat [27]. According to the survey, among Chinese patients, the prevalence rate in the north is significantly higher than that in the south, mainly because the north has sparse sunshine and cold climate [27]. Therefore, the skin pores, that is, the way from inside to outside, is blocked, and the internal heat does not dissipate, or even worse, thus forming a typical “Cold Envelops Heat” syndrome [27]. This syndrome is also closely related to the Xuan Fu theory [52]. Therefore, the treatment should be based on warming and dispersing methods as the main principles [27]. The outer cold dissipated, Xuan Fu unblocked, and the inner heat dissipated [52]. The classic clinical medicine *Ephedrae Herba*, Rhinoceros Horn and *Rehmanniae Radix* Decoction: *Ephedrae Herba Saposhnikoviae Radix* are pungent and warm to dispelled cold, Rhinoceros horn and *Rehmanniae Radix* clear heat and cool blood. Start with both internal and external channels at once, and get twice the result with half the effort [27].

To sum up, improving the heat, blood stasis, dryness and deficiency of blood is the ultimate goal, but regulating the viscera and the Qi of the body is the premise. We highly recommend that doctors consider the viscera factors in diagnosis and judge what organs have been damaged according to the patient’s skin lesion symptoms, accompanying symptoms, various physical functions and living habits. When the viscera function returns to normal, the blood will naturally be improved. For patients who are difficult to find the cause, the other three ideas may help doctors achieve twice the result with half the effort: For patients who onset or aggravation in winter, opening XuanFu could be considered; For patients living in a particularly cold place, warming method can be considered to eliminate “Cold Envelops Heat”; For patients with summer onset or aggravation and frequent recurrence, opening the meridians to reveal the hidden pathogenic factor could be considered.

## Treatments of psoriasis

### oral administration of Chinese medicine

Most of Chinese medicines used to treat psoriasis is decoction and patent medicine [55]. Therefore, in this part, we will elaborate on these two.

#### Decoction

The decoction used to treat psoriasis is very rich. Shibo Wang et al. [56] consulted literatures published in China in recent 20 years on the treatment of psoriasis by Chinese medicine s and the research type was clinical controlled trial, and the results showed that 327 decoctions were selected, involving 240 medicines. In addition, Qingjun Ma et al. [57] introduced 36 decoctions in the review of psoriasis prescription selection, of which 33 decoctions had been clearly studied, and a total of 2907 patients were treated, with an average effective rate of more than 92%. It can be seen that decoction has played a very high value in the process of treating psoriasis with Chinese medicine. Here, we also selected 15 decoctions in many literatures, Table 3. (Tables 3, 5, 6, 7 Only clinical data are summarized, and the medicine formulations are shown in the Additional file 2).

**Table 3:** Decoction

No	Decoction name	Syndrome types of patients	Number of cases	Efficiency (%)	Adverse reaction	References
1	Eliminate Ringworm and Destroy psoriasis Decoction	Blood Heat, Blood Dryness	500	99.4	No have	[58]
2	Eliminate Psoriasis Drink	Blood Heat, Blood Stasis	360	100	No have	[59]
3	Self-made Prescription	Blood Heat, Blood Stasis	135	98.6	No have	[60]
4	*Zaocys Bubali Cornu* Decoction	Four types are applicable	286	96.9	No have	[12]
5	Self-made and quick-acting Eliminate Psoriasis Powder	Blood Heat, Blood Dryness and Blood Stasis	296	95.6	No have	[61]
6	Clean Plague and Detoxify Decoction	Blood Heat	120	95	No have	[62]
7	*Taraxaci Herba* Decoction	Four types are applicable	118	94.9	Some patients had some discomfort such as dry mouth and nausea during the treatment, but there were no serious adverse reactions	[63]
8	Conquering Psoriasis Prescription	Blood Heat, Blood Deficiency	108	94.4	The symptoms of nausea, vomiting and poor appetite were observed in 2 patients	[64]
9	Cool Blood and Detoxify and Moisten Skin Decoction	Blood Heat, Blood Dryness and Blood Stasis	200	94	No have	[65]
10	Remove Stasis, Detoxify and Eliminate Speckle Decoction	Blood Heat, Blood Stasis	120	93	No have	[66]
11	Clear Heat and eliminate Psoriasis Decoction	Four types are applicable	120	92.5	No have	[23]
12	Eliminate Blood Stasis Decoction	Blood Stasis	125	92	No have	[67]
13	Cool Blood and Detoxify Decoction	Four types are applicable	106	92.5	No have	[68]
14	Five Detoxification Decoction	Blood Heat, Blood Dryness	100	90	No have	[69]
15	Remove Psoriasis Decoction	Blood Heat	206	94.7	No have	[70]

According to the contents in the above table, we believe that the most advantageous characteristics of decoction is: the application of medicines is very flexible. Doctors usually make some adjustments according to patients’ types and specific conditions, including medicine types and doses, such as Decoction 1 and 5. Here, we also summarize the commonly added medicines of each type or symptom characteristics, as shown in Table 4. This summary is not only based on the literatures we have consulted, including the above 15 decoctions, but also based on the experiences of many doctors such as Yanping Bai, Renkang Zhu and Tongyun Chen [14, 48, 71] on the application law of medicines. After clinical verification, they have no potential adverse reactions combined with other medicines, which can be used for reference. Moreover, this flexible method of adding and subtracting medicines is also the key to comprehensively regulate the functions of patients and accelerate the cure.

**Table 4:** Chinese medicines often added to various syndrome types or accompanying symptoms

Syndrome types or accompanying symptoms	Frequently added medicines
Blood Heat	*Arnebiae Radix*, *Sophorae Flos*, *Isatidis Folium*, *Forsythiae Fructus*, *Lonicerae Japonicae Flos*, *Coptidis Rhizoma*, *Radix Rhei et Rhizome*, *Rehmanniae Radix*
Blood Dryness	*Vespae Nidus*, *Adenophorae Radix*, *Dendrobii Caulis*, *Spatholobi Caulis*, *Sesami Semen Nigrum*, *Scrophulariae Radix*, *Phellodendri Chinensis Cortex*, *Rehmanniae Radix Praeparata*, *Anemarrhenae Rhizoma*
Blood Stasis	*Persicae Semen*, *Carthami Flos*, *Paeoniae Radix Rubra*, *Salviae Miltiorrhizae Radix et Rhizoma, Chuanxiong Rhizoma, Sparganii Rhizoma*, *Hirudo*, *Bombyx Batryticatus*
Blood Deficiency	*Angelicae Sinensis Radix*, *Paeoniae Radix Alba*, *Salviae Miltiorrhizae Radix et Rhizoma*, *Polygoni Multiflori Radix*, *Codonopsisradix*, *Asparagi Radix*
Lots of scales and sever pruritus	*Tribuli Fructus*, *Dioscore Aehypoglaucae Rhizoma*, *Pheretima*, *Zaocys, Cicadae Periostracum*, *Vespae Nidus*, *Clematidis Radix Et Rhizoma*, *Scorpio*, *Cnidii Fructus*
Mouth is dry and thirsty	*Ophiopogonis Radix*, *Scrophulariae Radix*, *Adenophorae Radix*
Qi deficiency	*Astragali Radix*, *Codonopsisradix*
Arthralgia	*Gentianae Macrophyllae Radix*, *Dictamni Cortex*, *Mori Ramulus*
Sore throat	*Sophorae Tonkinensis Radix et Rhizoma*, *Forsythiae Fructus*, *Arctii Fructus*, *Platycodonis Radix*
With damp heat	*Atractylodis Macrocephalae Rhizoma*, *Smilacis Glabrae Rhizoma*, *Atractylodis Rhizoma*, *Scutellariae Radix*
Anxiety and insomnia	*Margaritifera Concha*, *Ziziphi Spinosae Semen*
Upper limbs are severe	*Chuanxiong Rhizoma, Gentianae Macrophyllae Radix*
Lower limbs are severe	*Angelicae Pubescentis Radix*, *Achyranthis Bidentatae Radix*, *Stephaniae Tetrandrae Radix*
Head lesions are severe	*Crataegi Fructus*, *Campsis Flos*, *Smilacis Chinae Rhizoma*

#### Patent medicine

Although Chinese patent medicine is easy to carry and take, doctors can’t add or remove medicines according to specific symptoms. However, we found such an interesting phenomenon: Some doctors can take advantage of the characteristics of patent medicine to consolidate the treatment for patients who are in the late stage of treatment or have recovered. In the period of rapid onset, patients need to clear heat and cool blood quickly, so doctors will choose effective decoction. But such a long time will make the patient’s body consume too much and weak, so it is a better choice to use some patent medicines to consolidate in the later stage or after recovery. For example, when the famous dermatologist Guowei Xuan treated a patient, he applied the decoction used to cool blood and remove blood stasis in the early stage, and after the patient’s condition was stable, he applied the nourishing Six *Rehmanniae Radix* pill and *Salviae Miltiorrhizae Radix et Rhizoma* tablet to consolidate the condition, and finally achieved good curative effect [72]. If the cured patients are consolidated with patent medicine, it is an important means to reduce recurrence. Here, we briefly summarize 5 classic and commonly used patent medicines, Table 5.

**Table 5:** Chinese patent medicine

No	Chinese patent medicine name	Syndrome types of patients	Number of cases	Efficiency (%)	Adverse reaction	References
1	Eliminate Psoriasis Capsule	Blood Heat, Blood Dryness and Blood Stasis	2168	96.4	No serious adverse reactions	[73]
2	Compound Psoriasis Powder	Blood Heat	≥ 2000	≥ 90	No have	[74]
3	Eliminate Psoriasis Pill	Blood Heat, Blood Stasis	197	100	No have	[75]
4	Clear Psoriasis Pill	Four types are applicable; It is very suitable for late adjuvant treatment	145	99.3	No have	[76]
5	Yuan Family Psoriasis Pill	Blood Stasis	367	98.7	A few patients have the symptoms of anorexia, allergic dermatitis and slight decrease of leukocytes, but after symptomatic treatment, the patients will soon return to normal and can be treated normally	[77]

### External use of Chinese medicine

In addition to oral administration, external use of Chinese medicine is also a very common and important way. Because relieving dry skin and itching is not a quick thing, and external use of medicine can alleviate these symptoms from the outside [48].

#### Chinese medicine fumigation combined with phototherapy

Chinese medicine fumigation is a kind of external therapy to fumigate the body by using the steam generated from medicines to achieve the purpose of treatment [78]. Because it has a certain temperature and humidity, it has the advantages of transdermal absorption, improving blood circulation, promoting the regression of inflammation, and promoting the softening or elimination of inflammation [79]. Guihua Zhao et al. [80]. Once fumigated 200 patients with medicines such as *Smilacis Glabrae Rhizoma* and *Scutellariae Radix*, and the effective rate was 93.5%. Guoqing Yan et al. [81]. Once fumigated 40 patients with medicines such as *Spatholobi Caulis* and *Persicae Semen*, and the effective rate was 92.5%. Importantly, this method can promote sweating, and is highly consistent with sweating therapy of CM [82]. For example, Half of *Ephedrae Herba* and *Cinnamomi Ramulus* Geban Decoction, which can promote sweating, has been used in clinic, and after oral administration and fumigation with other prescriptions for 8 weeks, the effective rate is as high as 96% [83]. However, a recent meta-analysis on the comparison shows that Chinese medicine fumigation combined with phototherapy is better and safer than fumigation alone [87]. Therefore, here we summarize 10 effective methods of Chinese medicine fumigation combined with phototherapy in Table 6.

**Table 6:** Chinese medicine fumigation combined with phototherapy

No	Phototherapy	Syndrome types of patients	Number of cases	Efficiency (%)	Adverse reaction	References
1	NB-UVB (Narrow band-ultraviolet B)	Blood Heat, Blood Dryness	380	97.9	Two patients developed palpitation and chest tightness, which improved after lying flat, taking oxygen and resting for 10–20 min; After phototherapy, 7 patients had erythema or small blisters on their skin, and the symptoms disappeared after 3–7 days of suspension of treatment, local application of glucocorticoid ointment and wet compress with cold water	[88]
2	NB-UVB	Blood Heat, Blood Stasis, Blood Dryness	102	99	The skin of 15 patients had erythema and 12 patients had itchy and dry skin. The symptoms were alleviated after external application of moisturizer and adjustment of irradiation dose; All patients had skin pigmentation, recovered and stopped treatment for 1 month, and their skin color returned to normal	[89]
3	NB-UVB	Blood Heat, Blood Dryness	60	100	The skin of 3 patients was itchy and dry, and the symptoms were relieved after external application of moisturizer; All patients had skin pigmentation and gradually relieved after recovery	[90]
4	NB-UVB	Blood Heat	45	100	During fumigation, 2 patients had dizziness and 4 patients had dry mouth; Pigmentation spots appeared on the skin of all patients, but gradually relieved after stopping treatment	[91]
5	PUVA (Psoralen plus Ultraviolet A)	Blood Heat, Blood Stasis, Blood Dryness	40	100	Not mentioned	[92]
6	NB-UVB	Blood Heat, Blood Dryness	38	100	Skin erythema occurred in 11 cases, and the symptoms disappeared after external washing with *Calamina* and adjusting the dose of NB-UVB; Pigmentation spots appeared on the skin of all patients, but relieved after stopping treatment	[93]
7	NB-UVB	Blood Heat, Blood Dryness	66	98.5	No obvious abnormality is found (specific conditions are not mentioned)	[94]
8	NB-UVB	Blood Heat, Blood Dryness	51	98.04	No obvious abnormality is found (specific conditions are not mentioned)	[95]
9	NB-UVB	Blood Heat, Blood Stasis, Blood Dryness	50	98	Two patients had fever and chest tightness, and their symptoms improved after adjusting the fumigation temperature and time	[96]
10	NB-UVB	Blood Heat, Blood Dryness	76	97.4	Erythema occurred in 2 cases, pruritus in 6 cases and dry skin in 3 cases, but the symptoms disappeared after stopping NB-UVB irradiation; All patients had pigmentation spots on their skin after phototherapy, which were relieved after stopping treatment	[97]

#### Chinese medicine bath combined with phototherapy

In addition to fumigation, medicine bath is also a very common way. For example, Pengying Li et al. [98] once treated 100 patients with medicines such as *Portulacae Herba*, *Angelicae Sinensis Radix*, and the effective rate was 91%. Xiaokun Yan et al. [99] treated 45 patients with syndrome differentiation, and the effective rate was 95.56%. In addition, in addition to improving blood circulation and removing cutin, medicine bath also has some unique advantages over fumigation [98]. First, some patients are not suitable for fumigation, such as weak children, the elderly, in the late stage of treatment or blood deficiency syndrome, and patients with heart disease, hypertension and other diseases [100–102], and the medicine bath is a milder form [98]. Second, fumigation depends on fumigant, while medicine bath adopts immersion bath, which is more convenient for treatment [78, 98]. It is worth mentioning that doctors often fry the soup three times, the first two times for oral, and the last time for bathing. In a review on the methods of CM in the treatment of psoriasis, four methods of decoction and external washing of medicine residues were summarized, and 436 patients were treated, with an average effective rate of 94% [102]. Similarly, medicated bath is mostly combined with phototherapy, so it is summarized in Table 7.

**Table 7:** Chinese medicine bath combined with phototherapy

No	Phototherapy	Syndrome types of patients	Number of cases	Efficiency (%)	Adverse reaction	References
1	NB-UVB	Blood Heat, Blood Stasis, Blood Dryness	300	100	Not mentioned	[103]
2	NB-UVB	Blood Heat, Blood Stasis, Blood Dryness	196	100	Some patients had dry and itchy skin, and their symptoms improved after topical Vaseline cream; All patients had pigmentation spots on their skin after phototherapy, but gradually relieved after treatment	[104]
3	NB-UVB	Blood Heat	62	98.4	Skin itching and erythema occurred in 3 patients	[105]
4	NB-UVB	Blood Heat, Blood Dryness	57	98.2	Not mentioned	[106]
5	NB-UVB	Blood Heat, Blood Stasis, Blood Dryness	50	98	No obvious abnormality is found (specific conditions are not mentioned)	[107]
6	NB-UVB	Blood Heat, Blood Dryness	50	98	One patient had skin pruritus, one patient had burning sensation, and one patient had dry skin	[108]
7	NB-UVB	Blood Heat, Blood Dryness	75	97.3	After phototherapy, the skin of 2 patients was slightly dry and 3 patients had skin flushing and burning pain	[109]
8	NB-UVB	Blood Heat, Blood Stasis, Blood Dryness	56	96.4	After phototherapy, 10 patients had dry and itchy skin and 3 patients had light erythema. After external application of emollient and adjustment of irradiation dose, the symptoms disappeared	[110]
9	NB-UVB	Blood Heat, Blood Dryness	75	96	The skin of 3 patients was slightly dry and 5 patients had skin flushing and burning pain	[111]
10	NB-UVB	Blood Heat, Blood Stasis, Blood Dryness	60	95	Not mentioned	[112]

#### Local administration of Chinese medicine

Chinese medicine has many dosage forms for local treatment, such as ointment, liquid medicine and so on. The CM in the earliest literature on the treatment of psoriasis in China is a potion made of *Mylabris* and others [7]. Up to now, there are more and more kinds of medicines for local treatment. For example, Chunjiang Liu et al. [113] ed 703 patients with Conquer Psoriasis Ointment, and the effective rate is 99.7%; Dandan Tong et al. [114] treated 70 patients with *Indigo Naturalis* and *Sulfur* Ointment, and the effective rate is 100%; Zhenhan Sang et al. [115] treated 100 patients with Stubborn Psoriasis Liniment, and the effective rate is 99%.

### Others

In addition to the above methods, CM also has acupuncture, moxibustion, cupping, acupoint catgut embedding and other treatments, which are treasures of CM, and the curative effect is remarkable [116]. A review on the research progress of acupuncture and moxibustion in the treatment of psoriasis vulgaris summarizes more than 30 acupuncture and moxibustion methods that have been used in clinic, and shows that these methods have good curative effects [117]. However, these methods need to have a high degree of familiarity with human acupoints, proficiency in operation and accuracy, which is extremely difficult for doctors without theoretical knowledge and practical skills of CM [117]. Therefore, we do not recommend the promotion of this kind of method. On the contrary, we recommend cupping. Because Cupping is easier than acupuncture, and the risk is small [106]. An analysis of the effectiveness of combined cupping in the treatment of psoriasis vulgaris also shows that compared with conventional treatment, combined cupping has obvious advantages and is worthy of clinical promotion [118]. In addition, some folk prescriptions related to CM also have high curative effect, such as smell Chinese medicine smoke and Stings of bees [119, 120].

To sum up, the above summary methods are very good ones selected from a large number of literatures. They have been used in clinic. Not only the number of subjects is large, efficient and safe (the effective rates were higher than 90%), but also most of the medicines in the formula are high-frequency medicines for the treatment of psoriasis in clinic, which is very worthy of reference and promotion. (33 Chinese medicine s with more than 30 use frequency and the compatibility of medicines are summarized in the Additional file 3: Appendix Table S1, Additional file 4: Appendix Table S2.) However, the specific approach varies depending on the patient’s situation. We advocate oral administration combined external use of Chinese medicine. If possible, an additional cupping procedure will be more effective. Oral administration: For patients with rashes developing rapidly and accompanied with many symptoms, such as insomnia, we recommend decoction, and add or subtract medicines flexibly at any time; For patients who are in the late stage of treatment or have been cured, we recommend using patent medicines for consolidation treatment, which is also a good way to reduce the recurrence rate. External use: For young patients without contraindications such as heart disease, hypertension, anemia, ect, we recommend Chinese medicine fumigation; For the elderly or patients with contraindications, we recommend Chinese medicine bath or external washing of medicine residue of decoction; For patients with limited lesions and inconvenient conditions, the use of ointment and liquid medicine is also a good choice. Of course, the effect of fumigation and medicine bath combined with phototherapy is better, and there is basically no need to worry about adverse reactions: Pigmentation will be gradually relieved after treatment; Chest tightness, dizziness, poor breathing and other similar adverse reactions will be relieved after rest and adjusting the temperature of fumigation or bath; Moisturizing cream can be applied to itchy.

## Thoughts on the treatment of psoriasis with CM and suggestions on nursing

In addition to doctors implementing reasonable treatment plans, nursing and patients’ living habits are also an important part of treatment. Many doctors have some original thoughts in many years of experience, and their suggestions to patients also contain the wisdom of CM.

First, a reasonable diet. Don’t eat spicy or meat food at all can only guarantee that the patient will not recur for a while. But in the long run, the patient will lack antibodies and lead to the recurrence of such food as long as they eat a little food, and will also lack nutrition, zinc, manganese and other trace elements [121]. In addition, pepper, onion, ginger, garlic, coriander can also promote the secretion of sweat glands, so as to eliminate toxins [122]. In Chinese folk, it is useful to eat meat and blood food to lead harmful Qi from the body to skin for treatment. This wisdom can completely eliminate the hidden danger of “Hidden Pathogenic Factors” [121]. Second, get enough sleep. Studies have shown that a large part of the body’s healthy Qi comes from sleep. If the patient does not get enough sleep, the disease will not heal [123]. For example, many doctors give soothing medicines such as *Ziziphi Spinosae Semen* to improve sleep for patients with insomnia [63]. Third, keep the mood comfortable. Patients with psoriasis are under great pressure, which leads to depression and even mental illness. According to statistics, a certain proportion of patients with suicidal tendency [124]. Moreover, studies have found that patients with psoriasis usually show impairment of positive and neutral emotional memory, while negative emotional memory is relatively complete [125]. On the contrary, long-term depression, which is called “seven emotions internal injury” in CM, can also lead to heart and liver damage to trigger illness. At present, more and more doctors broke through the shackles and combine traditional therapy with psychotherapy, which have achieved gratifying results [126]. So, the patient’s emotions are very important for the treatment. Fourth, appropriate exercise. Some studies have also proved that sports sweating can alleviate psoriasis [84–86]. Moreover, exercise can also relieve mood, promote sweating, and can well help treatment.

In conclusion, the doctor’s order of “reasonable diet”, “ensuring sleep”, “maintaining a happy mood” and “proper exercise” necessary. Adding sleeping medicines to the prescription for insomnia patients, considering the factors of heart and liver for depressed patients, cooperating with emotional therapy when necessary, encouraging patients and enhancing patients’ confidence are of great value to the treatment, which is not be ignored to accelerate the cure and reduce the recurrence.

## Discussion

Although CM and modern medicine have different perspectives on psoriasis, they are also closely related to a certain extent. Firstly, in terms of classification, CM summarizes the Four-Type as the basic syndrome type of psoriasis. If the patient has pustules on the skin, it is considered that there is damp in the body [127]; If there are symptoms of erythroderma, it is considered that the situation of Blood Heat is too serious, resulting in Heat Toxicity [127]; If the patient has joint damage, it is considered that the pathogenic factors have involved the meridians [127]. However, there are also many literatures that clearly classify Four-Type into the vulgaris type within the scope of modern medicine, turn the pustular type into Damp Heat type, turn the erythroderma type into Toxic Heat type, and call the arthrosis type as Heat Resistance Meridian type [13, 128]. This is closely related to modern medicine, and we also believe that this classification is more rigorous. However, in fact, no matter what kind of statement, Four-Type is still the main in the classification of CM. Even the treatment principles of pustular type, erythroderma type and arthropathy type are also based on the treatment principles of Four-Type. In the 2017 edition of the consensus of CM experts on the treatment of psoriasis discussed by the dermatology branch of the Chinese society of CM, it is mentioned that the treatment principle of pustular psoriasis is to add remove dampness and detoxification on the basis of the treatment principle of Blood Heat, and the treatment principle of erythroderma type combines the treatment principle of Blood Heat and Blood Deficiency and the treatment principle of arthropathy type is to add unblock meridians on the basis of the treatment principle of Blood Stasis [13].For example, in the clinical experience of Doctor Kai Chen in the treatment of psoriasis, for patients with joint lesions, a decoction is used as the formula, and then the *Zaocys* and *Scorpio* with the effect of improving joint lesions are added [129]. In addition, the frequency of Four-Type is high, so we expound it in the main of the text. Second, with the development of modern research of CM, the pathogenesis and treatment principles of Four-Type are gradually combined with modern medical theory. For example, some scholars believe that there is a certain correlation between Four-Type and the degree of dermal papillary angiogenesis, and the positive expression of CD34 may become one of the indicators of Blood Heat [15]. Some studies have also confirmed that there are obvious differences in vascular morphology, blood flow velocity, erythrocyte aggregation, plasma endothelin content and Bcl-2 level in patients with Four-Type [130, 131]. More importantly, when doing clinical research on medicines (including decoction, patent medicine, single drug, etc.), researchers not only count the final effective rate, but also make a comparative study on the micro indicators of patients, such as hemorheology and immunity. For example, studies have proved that some Chinese medicine s for the treatment of Blood Heat can significantly reduce the quality of serum vascular endothelial growth factor, improve blood viscosity, regulate the dynamic balance of Th1/Th17 cytokines, etc. [15]. Some Chinese medicine s for the treatment of Blood Stasis can reduce the level of plasma endothelin, regulate blood lipid metabolism and apolipoprotein abnormalities [15]. In addition, CM believes that psoriasis can also be caused by the closure of Xuan Fu, Cold Envelopes Heat, resulting in the closure of skin pores [27, 51]. In this regard, the researchers also did experimental research on medicines with sweating effect such as Compound *Ephedrae Herba* Decoction, and the results showed that these medicines had a significant impact on the skin pathological tissue of mice, reflecting the relationship between the etiology of excessive epidermal appreciation in modern medicine and CM [132, 133]. Similarly, researchers not only study the micro indicators of patients, but also analyze the efficacy mechanism from the perspective of CM, and then analyze the modern research of Chinese medicine s. For example [40], *Rehmanniae Radix* and *Paeoniae Radix Rubra*, which have the function of clearing heat and cooling blood, can not only play the role of antipyretic, but also expand blood vessels, increase blood flow, improve microcirculation and enhance capillary density, so as to effectively improve the phenomenon of sieve hemorrhage in skin lesions, *Arnebiae Radix* can inhibit immune response, *Isatidis Folium* can increase leukocyte phagocytosis. *Persicae Semen* with the function of promoting blood circulation and removing blood stasis can reduce vascular resistance, increase blood flow, diminish inflammation and anti-allergy.

In addition to the study of blood, the study of viscera in CM has gradually deepened. For example, studies have confirmed that FAS and Fas-I in lung tissue are positive in skin and large intestine when lung injury occurs, which proves the connection between lung, large intestine and skin at the molecular level [38]. The CM theory that the heart dominates emotion and the liver is responsible for dredging emotion has gradually been proved in the research. For example, the study found that the occurrence of psoriasis is related to personality characteristics, and the personality characteristics of depression, introversion and stubbornness are more likely to occur [134]. The above also mentioned the importance of mental factors to psoriasis. Moreover, the skin is directly dominated by nerve fibers and extends to the outermost layer of the epidermis. These nerves can transmit a variety of information to the nerve center and stimulate the local inflammatory response of the skin to the injury [135]. More persuasively, a survey showed that 87.5% of patients with psoriasis had obvious pathological changes in their liver during liver biopsy [136]. In addition, CM believes that the kidney is the foundation of congenital, the root of Qi, the spleen and stomach is the foundation of postnatal and the source of Qi. Modern research shows that the strength of renal function is closely related to immunity, which is highly related to the pathogenesis of immune disorder in the field of modern medicine [55, 136]. For the theory of spleen and stomach, the intestinal flora, which has always been a research hotspot, is a very valuable proof. Not only have researchers recognized the close relationship between intestinal flora and spleen and stomach since the 1990s, but more and more studies have proved that the imbalance of intestinal flora is an important cause of psoriasis [137].

However, the above related research is still less and not thorough enough [55]. In the process of consulting the literature, we also found that most of the current clinical research literature still only expounds the pharmacodynamic mechanism from CM theory. More importantly, in order to improve the curative effect and shorten the treatment cycle, CM is not only treated in one or two ways, but often adopts a variety of ways, including the increasingly common way of combining Chinese and western medicine, such as decoction, medicine bath, combination of phototherapy, decoction, injection of western medicine, combination of acupuncture and moxibustion, etc. [102]. Although many of these methods have high effectiveness and safety, there are also some adverse reactions. Although this may be the patient’s own problem, because some studies have shown that the physique of patients with psoriasis is different, and smoking, diet and other reasons are closely related to side effects, attention must be paid to the potential adverse reactions of combined treatment methods [138, 139]. Therefore, it is very important to strengthen the modern research on the mechanism of efficacy, especially the compatibility of Chinese medicine s and the interaction between Chinese medicine s and western medicines. In addition, CM treatment of psoriasis also faces some other challenges. First, the treatment cycle is too long. Although there are many Chinese medicine s with rapid curative effect, patients still have to pay strong patience if they want to be cured completely. For example, the longest treatment cycle of some decoctions can reach 8 months [60]. This is a great test for patients with excessive psychological pressure. And there is still no patent medicine with definite curative effect and rapid effect like hormone replacement therapy [55]. Therefore, it is also imperative to study Chinese medicine s with rapid curative effect. Second, the dosage form needs to be reformed. At present, the traditional decoction and oral liquid are not easy to preserve, and the taking is cumbersome, which brings inconvenience to patients for a long time [55]. Although some Chinese medicine injections have been widely used, such as Compound *Salviae Miltiorrhizae Radix et Rhizoma* Injection [140], their comprehensive effect is not as good as decoction. In addition, many medicines are made from extracts of single Chinese medicine , such as *Daturaeflos*, *Tripterygii Radix* and *Peganum harmala L.* [141–143]. If the research can overcome the adverse reactions and make injections or other more dosage forms in the future, it will a big breakthrough.

## Conclusion and perspective

Compared with modern medicine, CM has unique advantages in the treatment of psoriasis, including the uniqueness of syndrome classification, the abundantly and flexibility of treatment ideas, as well as the efficiency and safety of treatment methods. We encourage doctors to treat patients learn from the process of CM: first, judge the syndrome type, then analyze the deeper causes affecting the blood, then implement specific treatment methods, and finally instruct patients to develop healthy living habits. Among them, the judgment type of syndrome can be based on the patient’s symptoms and characteristics, and it is advocated to ask the patient’s living environment and habits to assist in judgment; The analysis of etiology can also be based on the accompanying symptoms mentioned in the article, which are not only the experience of famous doctors, but also consistent with the theory of CM; The medicines mentioned in the text of the 5 (Treatments of Psoriasis) and summarized in the table have achieved surprising results in clinical trials, which can be directly referred to, and we suggest that doctors add or subtract drugs according to the specific situation of patients; It is worth noting that it is best to give medical orders to patients at any time, not just at the end of treatment.

The above are our views and suggestions according to the core content of traditional Chinese medicine in the treatment of psoriasis, and we deeply believe that its combination with modern medicine has high value. According to the content of modern research discussed in the discussion, we believe that it is necessary to increase the blood and organ pathological examination of patients, which not only is of great significance to judge the syndrome type and analyze the etiology, but also is a very breakthrough direction to promote modern research. We believe that in the process of Four-Type of changes, the research on blood indexes such as blood viscosity and the degree of dermal papilla angiogenesis, as well as the changes in regulating the dynamic balance of Th1/Th17 cytokines, is the key to the combination of CM “blood therapy” and modern medicine, and needs larger and deeper research. In terms of viscera, according to the theory of CM and the current situation of modern research, we put forward several directions that we think are of great research value: The mechanism of nutrition in the lung moistening the skin, the specific lesions of the liver and its impact on the blood during emotional disorders, the heart changes with the change of blood fever symptoms, the changes of the kidney under the premise of immune disorders, and the impact of the spleen and stomach on intestinal flora. It is worth mentioning that according to the theory of “XuanFu”, “Cold Envelope Heat” and the modern research of sweat method, the conclusion that sweating can alleviate psoriasis can be recognized. At the same time, we also encourage the exploration of factors such as living environment and living habits, and increasing factors such as temperature and diet in experimental modeling may be a promising new field. Moreover, because the lung is related to body fluid regulation, it seems to be a good direction to connect them with the research of lung. In terms of treatment, the use of prescriptions has been very mature, and decoction is the most recommended dosage form, fumigation or medicine bath combined with phototherapy is the most recommended external treatment method. However, in order to provide convenience for patients with inconvenient treatment, the development of injections and proprietary drugs should be developed. Among them, the top Chinese medicine s in the high-frequency medicines summarized in Additional file 3: Appendix Table S1, such as *Rehmanniae Radix*, *Paeoniae Radix Rubra*, *Glycyrrhizae Radix et Rhizoma*a, and *Daturaeflos*, *Tripterygii Radix* and *Peganum harmala L.* mentioned in the discussion have the very promising value of developing into injections, and extracting the effective components may become the hope of the emergence of specific Chinese medicines.

## Supplementary Information

**Additional file 1.** Analysis on the treatment and medication of each case of viscera treatment.

**Additional file 2.** Specific formula of decoction, patent medicine, fumigation prescription and medicine bath prescription.

**Additional file 3.** 33 kinds of Chinese medicines with more than 30 use frequency.

**Additional file 4.** The compatibility of medicines.

## Data Availability

Not applicable.
