# Supplementary material for: A review of Chinese medicine for the treatment of psoriasis: principles, methods and analysis
Source: Chin Med. 2021 Dec 20;16:138. doi: 10.1186/s13020-021-00550-y (PMC8686297; doi:10.1186/s13020-021-00550-y)
Supplement: Supplementary file 1 — Additional file 1. Analysis on the treatment and medication of each case of viscera treatment. [file 13020_2021_550_MOESM1_ESM.docx]

Supplementary Material 1. Patient situation, specific treatment principles and prescription analysis

| Viscera | Patient situation | Patient condition analysis | Medication analysis |
| --- | --- | --- | --- |
| Lung | Female, 34 years old.  The patient had a small amount of ringworm on his head three years ago and was treated by applying Chinese ointment, but the effect was not good. Later, because of eating too much mutton and spicy and greasy food in winter, the whole body itched, and ringworm pieces with clear boundaries, different sizes and different shapes appeared in the head, face, limbs, chest and abdomen. Seek medical treatment for a year, but the condition has not been controlled. | Mutton and other foods eaten by patients belong to hot food in traditional Chinese medicine, which is easy to produce heat. Therefore, the skin is caused by Blood Heat, which then affects the function of the lung. In addition, the symptoms of itching, dry skin and wrinkling are serious, so it is considered that the function of lung transportation of body fluid is affected. Therefore, the doctor judged that the patient lost the maintenance of body fluid and blood due to the poor function of lung upward Qi transmission. Therefore, the treatment principle is: unblock lung Qi, maintain blood, moisturize skin and relieve itching | A large dose of 1 can promote lung qi and make the function of lung transport body fluid normal, so as to spread nutrients to the skin; 2 and 1 cooperate with each other to make the lungs play the function of transmitting Qi upward and downward; 3 maintain blood, replenish body fluid, and balance Qi and blood, so as to maintain skin; 4 nourish body fluid and activate blood circulation; 5 relieve itching  All drugs cooperate with each other to play the functions of unblocking lung Qi, promoting blood circulation and improving circulation. |
| Large Intestine | Female, 23 years old.  Patients with psoriasis for many years, aggravated in winter and spring and alleviated in summer. Many treatments (including drugs and hot springs) have been used, and the curative effect is not good. At the end of autumn and the beginning of winter, the old disease relapsed. | At the beginning, doctors believed that the pathogenesis of patients was that dampness and heat were two pathogenic factors, which stagnated in the lung and the whole body, and affected the skin. Therefore, the treatment principle is: improve lung, clear heat and cool blood, remove moisture and maintain blood. However, after taking six doses of the prescription, the patient's skin lesions still did not reduce. At this time, the doctor considered that to remove the heat in the lung, improving the large intestine is the basis. Therefore, the treatment principle is to improve the function of large intestine conduction and discharge pathogenic factors from the intestine | The medicines in the initial prescription can clear away heat, cool blood and unblock lung Qi. *Rhei Radix et Rhizoma* and Cooked *Rhei Radix et Rhizoma* added in the third prescription have the function of catharsis, which can discharge the accumulated toxins in the large intestine, and then restore the function of the lung to normal. In addition, the last jujube can play a role in nourishing the body and maintaining blood. |
| Liver | Female, 48 years old.  Two years ago, without obvious inducement, the patient had a nail cap size rash at the bottom of both elbows, with white and gradually increasing scales. Later, the skin lesions spread to the head and trunk, accompanied by itching symptoms, aggravated in winter and alleviated in summer. One month before the initial diagnosis, the condition worsened, accompanied by poor appetite, dry mouth, bitter mouth, dry stool, upset and insomnia. | The patient has many rashes, red color, red tongue, yellow tongue coating, dry stool, belonging to Blood Heat; Due to the symptoms of upset and insomnia, doctors consider that the function of liver to dredge emotions is affected, so as to turn into heat. Therefore, the treatment principle is: Nourishing the liver, clearing away heat, cooling blood and detoxifying | In the Initial prescription: 1 cool blood and detoxifies, soothes liver Qi and clear away heat; 3 and 2 cooperate to detoxify; 4 regulate breath, promote blood circulation and cool blood; 5 dehumidification and antipruritic; 6 protect the stomach and reconcile all medicines  During the second diagnosis, due to the relief of pruritus, 5 were removed and 7 were added to enhance the effect of promoting blood circulation and detoxification.  Add 8 in the third diagnosis to strengthen the effect of promoting blood circulation.  The symptoms of Blood Heat were relieved during the forth diagnosis, so three blood cooling medicines were removed and 8 were added to play the role of moisturizing, drying and nourishing the body. |
| Heart | Male, 30 years old.  He has been ill for more than ten years. | The course of the disease was as long as ten years, and the pruritus was obvious, aggravated at night, and the scales rubbed and fell off. Therefore, the doctor judges that the pathogenesis is that the heat in the blood is too heavy to enter the heart, resulting in the dysfunction of the heart and liver, which is more serious. Therefore, the treatment principle is: clear the heat in the heart, cool the blood, nourish the liver, relieve itching and moisturize the skin | In the initial diagnosis, a large dose of 1 is used to clear away heat, cool blood and generate fluid. In particular, 2 with the function of clearing heat in the heart is added to the prescription；It can relieve itching, unblock meridians and dispel dampness; 4 can help the liver to dredge the mood of depression, 5 can remove the heat in the liver, and then play the role of relieving itching, and assist in regulating the function of the heart; In the later stage of treatment, the symptoms of blood fever gradually improved, so the drugs with strong heat clearing effect were removed to prevent damage to the body, and 6 were added to the prescription to strengthen the spleen, protect the stomach and dispel dampness. |
| Kidney | Male, 54 years old. Four years before the initial diagnosis, the patient had symptoms of increased dandruff due to damp in summer. No medication was used at that time. Later, his condition worsened, he went to a clinic and was diagnosed with psoriasis. The doctor gave oral western medicine and topical ointment for more than a month. Sometimes the symptoms were serious and sometimes alleviated. Later, the skin lesions gradually developed to the limbs and back. | The patient is older, has been ill for many years, and has blood stasis symptoms such as thick skin lesions, dark red and ecchymosis, and blood deficiency symptoms with thin and white tongue coating. Therefore, the doctor diagnosed the pathogenesis as: insufficient kidney qi, resulting in blood stasis and meridian block. Therefore, the treatment principle is: warming and tonifying the body, enhancing renal function, promoting blood circulation and removing blood stasis | 1 is used to warm the body and enhance renal function; 2 is used to promote blood circulation, remove blood stasis and smooth Qi; During the second diagnosis, the patient’s symptoms improved, so *Ginseng Radix et Rhizoma* and *Aconit Lateralis Radix Praeparata* were removed from the prescription, and 3 drugs with less heat and moderate nourishing power were added, and 4 drugs were added to generate fluid. |
| spleen and stomach | Male, 7 years old. The patient had dropwise papules and scales for one year. He had been treated with Cassiol ointment. Although the symptoms of scale shedding and itching were alleviated, they still occurred repeatedly. In addition, patients like to eat greasy, sweet food and lose control. | It can be inferred from the characteristics of skin lesions that the patient belongs to Blood Heat and it can be inferred from the patient's eating habits and other symptoms that the patient's spleen and stomach function is dysfunctional. In particular, redness and swelling of lips and dry stool are typical symptoms caused by indigestion of food in the stomach and obstruction of the function of spleen to transport body fluid, so as to turn into heat. Therefore, the treatment principle is: Help digestion and remove the heat accumulated in the spleen and stomach | Clearing heat and cooling blood, expelling the food accumulated in the intestines and stomach, so as to remove the heat in the spleen and stomach; Dissipate the heat hidden in the spleen; 3 clear heat in blood; 4. It is aromatic in nature and has a smooth spleen Qi; 5 clearing away heat and generating saliva; 6 clear away heat and reconcile all drugs |
