# Supplementary material for: A review of Chinese medicine for the treatment of psoriasis: principles, methods and analysis
Source: Chin Med. 2021 Dec 20;16:138. doi: 10.1186/s13020-021-00550-y (PMC8686297; doi:10.1186/s13020-021-00550-y)
Supplement: Supplementary file 2 — Additional file 2. Specific formula of decoction, patent medicine, fumigation prescription and medicine bath prescription. [file 13020_2021_550_MOESM2_ESM.docx]

Supplementary Material 2. Formula of Decoction, Patent medicine, Fumigation and Bath

Decoction:

| 1 | *Cicadae Periostracum*, *Bombyx Batryticatus*, *Scolopendra*, *Scorpio* (Grind into powder), *Campsis Flos*, *Serpentis Periostracum*, Dry stems and leaves of *Maytenus hookeri Loes.*, *Salviae Miltiorrhizae Radix et Rhizoma*, *Saposhnikoviae Radix*, *Scutellariae Radix*, *Sophorae Flavescentis Radix*,   1. Blood Heat: Add *Sophorae Flos*, *Imperatae Rhizoma*, *Isatidis Folium* 2. Blood Dryness: Add *Angelicae Sinensis Radix*, *Ophiopogonis Radix*, *Asparagi Radix* 3. There are pustules: Add *Atractylodis Rhizoma*, *Phellodendri Chinensis Cortex*, *Lonicerae Japonicae Flos* 4. Skin redness and swelling: Add *Fritillariae Cirrhosae Bulbus*, *Bubali Cornu*, *Moutan Cortex*, *Gardeniae Fructus* 5. Spleen deficiency: Add Scorched *Atractylodis Macrocephalae Rhizoma*, *Poria* 6. Severe itching: Add *Dictamni Cortex*, *Tribuli Fructus* 7. Women have less menstruation or blood clots: Add *Leonur Iherba*, *Carthami Flos* |
| --- | --- |
| 2 | *Rehmanniae Radix*, *Angelicae Sinensis Radix*, *Phellodendri Chinensis Cortex*, *Scrophulariae Radix*, *Arnebiae Radix*, *Salviae Miltiorrhizae Radix et Rhizoma*, *Paeoniae Radix Rubra*, *Glycyrrhizae Radix et Rhizoma*, *Isatidis Radix*, *Smilacis Glabrae Rhizoma*   1. Blood Heat: Add *Bistortae Rhizoma* 2. Blood Stasis: Add *Spatholobi Caulis* 3. Dry mouth and constipation: Add *Rhei Radix et Rhizoma*, *Forsythiae Fructus* 4. Severe head lesions: Add *Crataegi Fructus* 5. Severe itching: Add *Zaocys*, *Vespae Nidus* |
| 3 | *Rehmanniae Radix Praeparata*, *Chuanxiong Rhizoma*, *Paeoniae Radix Alba*, *Angelicae Sinensis Radix*, *Corni Fructus*, *Saposhnikoviae Radix*, *Cicadae Periostracum*, *Tribuli Fructus*, *Dictamni Cortex*, *Glycyrrhizae Radix et Rhizoma*   1. Constipation: Add *Rhei Radix et Rhizoma* 2. Large areas of erythema and the color of the tongue and face is red (Blood Heat): Add *Rehmanniae Radix*, *Scutellariae Barbatae Herba* 3. Long course of illness and the color of the lesion is dark red (Blood Stasis): Add *Salviae Miltiorrhizae Radix et Rhizoma*, *Eupolyphaga Steleophaga* |
| 4 | *Zaocys*, *Bubali Cornu* (Fried in advance), *Moutan Cortex*, *Bombyx Batryticatus*, *Cicadae Periostracum*, *Dictamni Cortex*, *Angelicae Sinensis Radix*, *Scolopendra*, *Smilacis Glabrae Rhizoma* ^1^, *Bistortae Rhizoma*  (1) Blood Heat: Add *Lonicerae Japonicae Flos*, *Sophorae Flos*, *Rehmanniae Radix*  (2) Blood Dryness: Add *Cannabis Fructus*, *Polygoni Multiflori Radix*, *Rehmanniae Radix*  (3) Blood Stasis: Add *Salviae Miltiorrhizae Radix Et Rhizoma*, *Gleditsiae Spina*  (4) Blood Deficiency: Remove 1 and add *Polygoni Multiflori Caulis*, *Pseudostellariae Radix*  (5) Blood Heat and there is moisture in the body: Add *Lonicerae Japonicae Flos*, *Clematidis Radix Et Rhizoma*, *Hedyotis Diffusae Herba*  (6) Severe head lesions: Add *Puerariae Lobatae Radix*, *Chrysanthemi Indici Flos*  (7) Severe itching: Add *Tribuli Fructus*, *Sophorae Flavescentis Radix* |
| 5 | *Smilacis Glabrae Rhizoma*, *Moutan Cortex* ^2^, *Scutellariae Radix*, *Arnebiae Radix* ^1^, *Rehmanniae Radix* ^2^, *Scrophulariae Radix* ^2^, *Carthami Flos* ^3^, *Bubali Cornu* Powder ^2^, *Astragali Radix* ^3^, *Angelicae Sinensis Radix* ^3^, *Dictamni Cortex*, *Lonicerae Japonicae Flos* ^1^, *Scutellariae Barbatae Herba* ^1^, *Polygoni Multiflori Radix* ^3^, *Scolopendra*, *Zaocys*, *Hedyotis Diffusae Herba* ^1^  （1） Blood Heat: Increase the dosage of 1  （2）Blood Dryness: Increase the dosage of 2  （3）Blood Stasis: Increase the dosage of 3 |
| 6 | *Gypsum Fibrosum* (Fried in advance), *Anemarrhenae Rhizoma*, *Rehmanniae Radix*, *Moutan Cortex*, *Coptidis Rhizoma*, *Scutellariae Radix*, *Gardeniae Fructus*, *Lophatheri Herba*, *Scrophulariae Radix*, *Paeoniae Radix Rubra*, *Forsythiae Fructus*, *Glycyrrhizae Radix et Rhizoma*  (1) Dry mouth: Add *Asparagi Radix*, *Ophiopogonis Radix*  (2) There is exudation in the skin: Add *Sophorae Flavescentis Radix*, *Dictamni Cortex*  (3) Later, the flush faded and erythema appeared: Add *Smilacis Glabrae Rhizoma*, *Sophorae Flos*  (4) Sore throat with fever and cough: Add *Houttuyniae Herba*, *Isatidis Radix*, *Taraxaci Herba* |
| 7 | *Taraxaci Herba*, *Rehmanniae Radix*, *Phragmitis Rhizoma*, *Dictamni Cortex*, *Isatidis Radix*, *Forsythiae Fructus*, *Moutan Cortex*, *Ophiopogonis Radix*, *Scrophulariae Radix*, *Lonicerae Japonicae Flos*, *Sophorae Tonkinensis Radix et Rhizoma*, *Platycodonis Radix*, *Glycyrrhizae Radix et Rhizoma*  （1）The rash is dark brown (Blood Stasis): Add *Carthami Flos*, *Persicae Semen*  （2）Fever, irritability and thirst (Blood Heat): Add *Gypsum Fibrosum*, *Anemarrhenae Rhizoma*  （3）Severe itching: Add *Cnidii Fructus*, *Vespae Nidus*  （4）Anxiety and insomnia: Add Frying *Ziziphi Spinosae Semen*, *Margaritifera Concha*  （5）Fatigue and weakness (Partial to Blood Deficiency): Add *Astragali Radix*, *Angelicae Sinensis Radix* |
| 8 | （1）Blood Heat: Conquering Psoriasis Prescription 1  *Smilacis Glabrae Rhizoma*, *Lonice Raejaponicae Caulis*, *Sophorae Tonkinensis Radix et Rhizoma*, *Isatidis Radix*, *Bistortae Rhizoma*, *Dictamni Cortex*, *Clematidis Radix et Rhizoma*, *Glycyrrhizae Radix et Rhizoma*  （2）Blood Deficiency: Conquering Psoriasis Prescription 2  *Rehmanniae Radix*, *Salviae Miltiorrhizae Radix et Rhizoma*, *Scrophulariae Radix*, *Cannabis Fructus*, *Isatidis Folium*, *Sophorae Tonkinensis Radix et Rhizoma*, *Dictamni Cortex*, *Bistortae Rhizoma*, *Forsythiae Fructus* |
| 9 | *Spatholobi Caulis*, *Imperatae Rhizoma*, *Smilacis Glabrae Rhizoma*, *Sophorae Tonkinensis Radix et Rhizoma*, *Trichosanthis Radix*, *Arnebiae Radix*, *Rehmanniae Radix*, *Scrophulariae Radix*, Scorched *Crataegi Fructus*, *Rubiae Radix et Rhizoma*  （1）Blood Heat: Add *Sophorae Flos*, *Lonicerae Japonicae Flos*, *Moutan Cortex*  （2）Blood Dryness: Add *Angelicae Sinensis Radix*, *Ophiopogonis Radix*, *Mume Fructus*  （3）Blood Stasis: Add *Chuanxiong Rhizoma*, *Persicae Semen*, *Carthami Flos*  （4）Blood Heat and there is moisture in the body: Add *Dioscore Aehypoglaucae Rhizoma*, *Coicis Semen*, *Dioscoreae Rhizoma*  （5）Blood Heat is very serious: Add *Coptidis Rhizoma*, *Rhei Radix et Rhizoma*, *Phellodendri Chinensis Cortex* |
| 10 | *Rehmanniae Radix* ^2^, *Scrophulariae Radix*, *Angelicae Sinensis Radix*, *Sparganii Rhizoma*, *Salviae Miltiorrhizae Radix et Rhizoma*, *Arnebiae Radix* ^1^, *Paeoniae Radix Rubra* ^3^, *Spatholobi Caulis*, *Angelicae Dahuricae Radix*, *Polygoni Cuspidati Rhizoma et Radix* ^1^, *Asparagi Radix*, *Ophiopogonis Radix*, *Trichosanthis Radix*, *Indigo Naturalis* ^1^, *Tripterygii Radix*  (1) Progressive stage: Dosage of 1 is large  (2) Quiescence and regression: Replace 2 with *Rehmanniae Radix Praeparata* and replace 3 with *Paeoniae Radix Alba* |
| 11 | *Lonicerae Japonicae Flos* (Fried after), *Dictamni Cortex*, *Schizonepetae Herba*, *Saposhnikoviae Radix*, *Spatholobi Caulis*, *Euphorbiae Ebracteolatae Radix*, Stir-frying *Manis Squama*, *Arnebiae Radix*, *Angelicae Dahuricae Radix*, *Forsythiae Fructus*, *Carthami Flos*, *Mori Cortex*, *Cicadae Periostracum*, *Gleditsiae Spina*, *Zaocys*, *Rehmanniae Radix*, *Glycyrrhizae Radix et Rhizoma*   1. Blood Heat: Add *Bubali Cornu*, *Scrophulariae Radix*, *Moutan Cortex* 2. Blood Stasis: Add *Salviae Miltiorrhizae Radix Et Rhizoma*, *Sparganii Rhizoma*, *Cicadae Periostracum*, *Gleditsiae Spina*, *Zaocys*, *Paeoniae Radix Rubra*, *Rehmanniae Radix*, *Glycyrrhizae Radix et Rhizoma* 3. Blood Deficiency: Add *Polygoni Multiflori Radix*, *Angelicae Sinensis Radix*, *Rehmanniae Radix Praeparata* 4. Blood Heat and there is moisture in the body: Add *Coicis Semen*, *Atractylodis Rhizoma*, *Smilacis Glabrae Rhizoma* 5. The scales are thick and not easy to fall off: Add *Salviae Miltiorrhizae Radix Et Rhizoma*, *Astragali Radix*, *Angelicae Sinensis Radix* 6. Severe itching: Add *Sophorae Flavescentis Radix*, *Tribuli Fructus*   （7）The plaque is hard and thick: Add *Sargassum*, *Laminariae Thallus Eckloniae Thallus* |
| 12 | *Angelicae Sinensis Radix*, *Persicae Semen*, *Carthami Flos*, *Paeoniae Radix Rubra*, *Chuanxiong Rhizoma*, *Salviae Miltiorrhizae Radix et Rhizoma*, *Rehmanniae Radix*, *Arnebiae Radix*, *Hedyotis Diffusae Herba* |
| 13 | *Rehmanniae Radix*, *Paeoniae Radix Rubra*, *Moutan Cortex*, *Imperatae Rhizoma*, *Smilacis Glabrae Rhizoma*, *Dictamni Cortex*, *Clematidis Radix et Rhizoma*, *Bistortae Rhizoma*, *Scutellariae Barbatae Herba*, *Polygoni Cuspidati Rhizoma et Radix*, *Hedyotis Diffusae Herba*, *Glycyrrhizae Radix et Rhizoma*   1. It is in progress and has obvious erythema (Blood Heat): Add *Gypsum Fibrosum*, *Anemarrhenae Rhizoma* 2. It's in a quiescent period and lesions are hypertrophic (Blood Stasis): Add *Salviae Miltiorrhizae Radix et Rhizoma*, *Sparganii Rhizoma*, *Curcumae Rhizoma* 3. It's in a period of extinction and the color of red spots are light (Blood Deficiency): Add *Angelicae Sinensis Radix*, *Polygoni Multiflori Radix* 4. Sore throat: Add *Scrophulariae Radix*, *Isatidis Radix* 5. The itching is obvious: Add *Sophorae Flavescentis Radix*, *Tribuli Fructus* 6. Constipation: Add *Isatidis Folium* |
| 14 | *Chrysanthemi Indici Flos* ^1^, *Taraxaci Herba*, *Rehmanniae Radix*, *Gardeniae Fructus*, *Scrophulariae Radix*, *Isatidis Radix*, *Angelicae Sinensis Radix*, *Paeoniae Radix Rubra*, *Trichosanthis Radix*, *Puerariae Lobatae Radix*, *Tribuli Fructus*, *Smilacis Glabrae Rhizoma*, *Violae Herba* ^1,^ *Fritillariae Cirrhosae Bulbus*, *Glycyrrhizae Radix et Rhizoma*  (1) Severe itching：Add *Dictamni Cortex*  (2) Dry skin and more scales: Add *Spatholobi Caulis*, *Polygoni Multiflori Radix*  (3) Loose stool and eat less: Remove 1 and add Dioscoreae Rhizoma, Scorched *Crataegi Fructus* |
| 15 | *Rehmanniae Radix*, *Scrophulariae Radix*, *Ophiopogonis Radix*, *Coptidis Rhizoma* ^1^, *Scutellariae Radix* ^1^, *Lonicerae Japonicae Flos*, *Isatidis Folium*, *Saposhnikoviae Radix*, *Angelicae Sinensis Radix*, *Salviae Miltiorrhizae Radix et Rhizoma*, *Eupolyphaga Steleophaga* ^1^, Jujube   1. Severe Blood Heat: Add *Gypsum Fibrosum*, Gypsum Fibrosum Powder, *Indigo Naturalis* Powder 2. Blood Dryness: Remove 1, reduce the dose of 2 and add *Polygoni Multiflori Radix*, *Dictamni Cortex* 3. Damp heat: Add *Atractylodis Rhizoma*, *Xanthii Fructus* 4. Severe head lesions: Add *Angelicae Dahuricae Radix*, *Puerariae Lobatae Radix* 5. Severe skin lesions of lower limbs: Add *Cyathulae Radix* 6. Dry stool: Add *Rhei Radix et Rhizoma*, *Gardeniae Fructus* 7. Severe itching: Add *Dictamni Cortex* 8. Spleen deficiency: Add *Poria* 9. Elderly, children, frail patients: Appropriate dose reduction |

Patent medicine:

| 1 | *Scorpio*, *Scolopendra*, *Mylabris*, *Bungarus Parvus*, *Bovis Calculus*, *Manis Squama*, Bud of *Sophora japonica L.*, et |
| --- | --- |
| 2 | *Cordyceps*, *Zaocys*, *Indigo Naturalis*, *Saposhnikoviae Radix*, *Isatidis Radix*, *Bovis Calculus Artifactus*, *Gentianae Macrophyllae Radix*, *Angelicae Dahuricae Radix*, *Cicadae Periostracum*, *Chrysanthemi Indici Flos*, *Hedyotis Diffusae Herba*, *Rehmannia*e *Radix*, *Sophorae Flos*, *Lonicerae Japonicae Flos*, *Smilacis Glabrae Rhizoma*, *Dictamni Cortex*, *Mume Fructus*, *Glycyrrhizae Radix et Rhizoma*, *Arctii Fructus*, *Arnebiae Radix*, *Imperatae Rhizoma*, *Euphorbiae Ebracteolatae Radix*, et |
| 3 | *Smilacis Glabrae Rhizoma*, *Isatidis Folium*, *Isatidis Radix*, *Rhei Radix et Rhizoma*, *Lycii Cortex*, *Rehmanniae Radix*, *Scrophulariae Radix*, *Paeoniae Radix Rubra*, *Arnebiae Radix*, *Lonicerae Japonicae Flos*, *Arctii Fructus*, *Cicadae Periostracum*, *Glycyrrhizae Radix et Rhizoma*, *Psoraleae Fructus*, *Carthami Flos*, fruit of *Ficus carica L.* |
| 4 | *Smilacis Glabrae Rhizoma*, *Moutan Cortex*, *Anemarrhenae Rhizoma*, *Scrophulariae Radix*, *Sophorae Flavescentis Radix*, *Ophiopogonis Radix*, *Rehmanniae Radix*, *Lonicerae Japonicae Flos*, *Salviae Miltiorrhizae Radix et Rhizoma*, *Cicadae Periostracum*, *Saposhnikoviae Radix*, *Prunellae Spica*, *Chuanxiong Rhizoma*, *Schizonepetae Herba*, *Forsythiae Fructus*, *Glycyrrhizae Radix et Rhizoma* |
| 5 | *Salviae Miltiorrhizae Radix et Rhizoma*, *Manis Squama*, *Myrrha*, *Curcumae Rhizoma*, *Bistortae Rhizoma*, *Scolopendra*, *Scrophulariae Radix*, et |

Chinese medicine Fumigation Combined with Phototherapy:

| 1 | *Phryma leptostachya L. subsp. asiatica (Hara) Kitamura*, *Platycladi Cacumen*, *Gleditsiae Sinensis Fructus*, *Portulacae Herba*, *Sophorae Flavescentis Radix*, *Dictamni Cortex*, *Phellodendri Chinensis Cortex*, *Kochiae Fructus* |
| --- | --- |
| 2 | *Rehmanniae Radix*, *Moutan Cortex*, *Paeoniae Radix Rubra*, *Dictamni Cortex*, *Kochiae Fructus*, *Saposhnikoviae Radix*、*Spatholobi Caulis*、*Polygoni Multiflori Caulis*、*Salviae Miltiorrhizae Radix et Rhizoma* |
| 3 | Blood Heat: *Phellodendri Chinensis Cortex*, *Sophorae Flavescentis Radix*, *Kochiae Fructus*, *Dictamni Cortex*, *Rhei Radix et Rhizoma*, et  Blood Dryness: *Arnebiae Radix*, *Rehmanniae Radix*, *Sophorae Flavescentis Radix*, *Lonicerae Japonicae Flos*, *Moutan Cortex*, et |
| 4 | *Rhei Radix et Rhizoma*, *Senecionis Scandentis Hebra*, *Hydnocarpus anthelmintica Pierre*, *Smilacis Glabrae Rhizoma*, *Kochiae Fructus*, *Cnidii Fructus*、*Phellodendri Chinensis Cortex*、*Schizonepetae Herba*、*Chrysanthemi Indici Flos*, *Sophorae Flavescentis Radix* |
| 5 | *Sophorae Flavescentis Radix*, *Kochiae Fructus*, *Dictamni Cortex*, *Salviae Miltiorrhizae Radix et Rhizoma*, *Isatidis Radix*, *Cnidii Fructus*, *Chrysanthemi Flos*, *Angelicae Sinensis Radix* |
| 6 | *Poria*, *Kochiae Fructus*, *Angelicae Sinensis Radix*, *Dictamni Cortex*, *Cynanchi Paniculati Radix et Rhizoma*, *Cnidii Fructus*, *Xanthii Fructus*, *Stemonae Radix*, *Cleistocalycis Operculati Cortex*, *Tribuli Fructus*, *Rhei Radix et Rhizoma* |
| 7 | *Lonicerae Japonicae Flos*, *Taraxaci Herba*, *Angelicae Sinensis Radix*, *Rehmanniae Radix*, *Sophorae Flavescentis Radix*, *Chuanxiong Rhizoma*, *Saposhnikoviae Radix*, *Cynanchi Paniculati Radix et Rhizoma*, *Dictamni Cortex*, *Kochiae Fructus* |
| 8 | *Platycladi Cacumen*, *Natrii Sulfas*, *Alumen*, *Chrysanthemi Indici Flos*, *Dictamni Cortex*, *Sophorae Flavescentis Radix*, *Lonicerae Japonicae Flos*, *Lycii Cortex* |
| 9 | *Xanthii Fructus*, *Cinnamomi Ramulus*, *Cnidii Fructus*, *Polygoni Multiflori Caulis*, *Angelicae Sinensis Radix*, *Gentianae Macrophyllae Radix*, *Kochiae Fructus*, *Smilacis Glabrae Rhizoma*, *Dictamni Cortex*, et |
| 10 | *Platycladi Cacumen*, *Alumen*, *Natrii Sulfas*, *Chrysanthemi Indici Flos*, *Dictamni Cortex*, *Sophorae Flavescentis Radix*, *Lonicerae Japonicae Flos*, *Lycii Cortex* |

Chinese medicine Bath Combined with Phototherapy:

| 1 | *Kochiae Fructus*, *Dictamni Cortex*, *Salviae Miltiorrhizae Radix et Rhizoma*, *Saposhnikoviae Radix*, *Cnidii Fructus* |
| --- | --- |
| 2 | *Cnidii Fructus*, *Sophorae Flavescentis Radix*, *Polygoni Cuspidati Rhizoma et Radix*, *Salviae Miltiorrhizae Radix et Rhizoma*, *Atractylodis Rhizoma*, *Cynanchi Paniculati Radix et Rhizoma*, *Zanthoxyli Pericarpium*, Patriniae, *Portulacae Herba* |
| 3 | *Phellodendri Chinensis Cortex*, *Angelicae Sinensis Radix*, *Psoraleae Fructus*, *Galla Chinensis*, *Curcumae Longae Rhizoma* |
| 4 | *Salviae Miltiorrhizae Radix et Rhizoma*, *Angelicae Sinensis Radix*, *Kochiae Fructus*, *Dictamni Cortex*, *Phellodendri Chinensis Cortex*, *Prunellae Spica*, *Isatidis Folium*, *Smilacis Glabrae Rhizoma* |
| 5 | *Kochiae Fructus*, *Sophorae Flavescentis Radix*, *Atractylodis Rhizoma*, *Polygoni Cuspidati Rhizoma et Radix*, *Salviae Miltiorrhizae Radix et Rhizoma*, Patriniae, *Zanthoxyli Pericarpium*, *Portulacae Herba*, *Cynanchi Paniculati Radix et Rhizoma*, *Stemonae Radix* |
| 6 | *Chrysanthemi Indici Flos*, *Phellodendri Chinensis Cortex*, *Kochiae Fructus*, *Cnidii Fructus*, *Lonicerae Japonicae Flos*, *Angelicae Dahuricae Radix*, *Acori Tatarinowii Rhizoma* |
| 7 | *Sophorae Flavescentis Radix*, *Chrysanthemi Indici Flos*, *Violae Herba*, *Lonicerae Japonicae Flos*, *Cnidii Fructus*, *Cynanchi Paniculati Radix et Rhizoma*, *Glycyrrhizae Radix et Rhizoma* |
| 8 | *Polygoni Multiflori Radix*, *Mume Fructus*, *Rehmanniae Radix*, *Spatholobi Caulis*, et |
| 9 | *Sophorae Flavescentis Radix*, *Chrysanthemi Indici Flos*, *Violae Herba*, *Cnidii Fructus*, *Lonicerae Japonicae Flos*, et |
| 10 | *Arnebiae Radix*, *Spatholobi Caulis*, *Salviae Miltiorrhizae Radix et Rhizoma*, *Paeoniae Radix Rubra*, *Lonicerae Japonicae Flos*, *Isatidis Folium*, *Polygoni Cuspidati Rhizoma et Radix*, *Rehmanniae Radix*, *Sparganii Rhizoma*, *Curcumae Rhizoma*, *Smilacis Glabrae Rhizoma*, *Dictamni Cortex*, *Tribuli Fructus*, *Angelicae Sinensis Radix* |
