# Supplementary material for: A review of Chinese medicine for the treatment of psoriasis: principles, methods and analysis
Source: Chin Med. 2021 Dec 20;16:138. doi: 10.1186/s13020-021-00550-y (PMC8686297; doi:10.1186/s13020-021-00550-y)
Supplement: Supplementary file 3 — Additional file 3. 33 kinds of Chinese medicines with more than 30 use frequency. [file 13020_2021_550_MOESM3_ESM.docx]

Appendix Table 1 33 Kinds of Chinese Medicines with More Than 30 Use Frequency

| Chinese medicine | Frequency | Chinese medicine | Frequency | Chinese medicine | Frequency |
| --- | --- | --- | --- | --- | --- |
| *Rehmanniae Radix* | 204 | *Sophorae Flos* | 66 | *Forsythiae Fructus* | 46 |
| *Paeoniae Radix Rubra* | 163 | *Carthami Flos* | 65 | *Saposhnikoviae Radix* | 45 |
| *Glycyrrhizae Radix et Rhizoma* | 158 | *Chuanxiong Rhizoma* | 58 | *Bubali Cornu* | 45 |
| *Moutan Cortex* | 143 | *Scrophulariae Radix* | 57 | *Isatidis Folium* | 44 |
| *Angelicae Sinensis Radix* | 143 | *Isatidis Radix* | 56 | *Cicadae Periostracum* | 42 |
| *Smilacis Glabrae Rhizoma* | 134 | *Zaocys* | 51 | *Rehmanniae Radix Praeparata* | 41 |
| *Salviae Miltiorrhizae Radix et Rhizoma* | 134 | *Imperatae Rhizoma* | 51 | *Curcumae Rhizoma* | 39 |
| *Dictamni Cortex* | 128 | *Persicae Semen* | 49 | *Ophiopogonis Radix* | 36 |
| *Arnebiae Radix* | 113 | *Scutellariae Radix* | 47 | *Rhei Radix et Rhizoma* | 33 |
| *Hedyotis Diffusae Herba* | 104 | *Paeoniae Radix Alba* | 47 | *Sparganii Rhizoma* | 32 |
| *Lonicerae Japonicae Flos* | 88 | *Sophorae Flavescentis Radix* | 46 | *Vespae Nidus* | 30 |
