# Supplementary material for: A review of Chinese medicine for the treatment of psoriasis: principles, methods and analysis
Source: Chin Med. 2021 Dec 20;16:138. doi: 10.1186/s13020-021-00550-y (PMC8686297; doi:10.1186/s13020-021-00550-y)
Supplement: Supplementary file 4 — Additional file 4. The compatibility of medicines. [file 13020_2021_550_MOESM4_ESM.docx]

Appendix Table 2 the compatibility of medicines

| **Effect** | **Medicine** |
| --- | --- |
| Clearing Heat + Cooling blood | 1 *Rehmanniae Radix* + *Imperatae Rhizoma*  2 *Rehmanniae Radix* + *Bubali Cornu*  3 *Rehmanniae Radix* + *Isatidis Folium*  4 *Rehmanniae Radix* + *Isatidis Radix*  5 *Rhei Radix et Rhizoma* + *Glycyrrhizae Radix et Rhizoma*  6 *Rehmanniae Radix* + *Moutan Cortex* + *Arnebiae Radix* + *Paeoniae Radix Rubra* |
| Tonifying blood + Activating blood | 1 *Angelicae Sinensis Radix* + *Carthami Flos* + *Paeoniae Radix Rubra*  2 *Paeoniae Radix Alba* + *Rehmanniae Radix Praeparata* + *Chuanxiong Rhizoma* |
| Clearing blood+ Removing dampness and relieving itching | *Smilacis Glabrae Rhizoma* + *Dictamni Cortex* |
| Clearing heat+ Activating blood | *Hedyotis diffusa* + *Salviae Miltiorrhizae Radix et Rhizoma* + *Spatholobi Caulis* |
| Clearing heat and cooling blood + Hemostasis | *Isatidis Radix* + *Isatidis Folium* + *Sophorae Flos* + *Imperatae Rhizoma* |
| Clearing heat + Removing rash | *Sophorae Flavescentis Radix* + *Cicadae Periostracum* + *Saposhnikoviae Radix* + *Zaocys* |
| Promoting blood circulation and removing blood stasis + Smoothing Qi | 1 *Angelicae Sinensis Radix* + *Chuanxiong Rhizoma*  2 *Carthami Flos* + *Persicae Semen* + *Sparganii Rhizoma* + *Curcumae Rhizoma* |
| Clearing heat and cooling blood + Diarrhea + Moistening lung and generating fluid | *Scutellariae Radix* + *Forsythiae Fructus* + *Rhei Radix et Rhizoma* + *Vespae Nidus* + *Ophiopogonis Radix* + *Bubali Cornu* + *Scrophulariae Radix* |
